# An unknown collection of lizards from Afghanistan

**DOI:** 10.3897/zookeys.843.29420

**Published:** 2019-05-09

**Authors:** Daniel Jablonski, Aleksandar Urošević, Marko Andjelković, Georg Džukić

**Affiliations:** 1 Department of Zoology, Comenius University in Bratislava, Ilkovičova 6, Mlynská dolina, 842 15 Bratislava, Slovakia Comenius University in Bratislava Bratislava Slovakia; 2 University of Belgrade, Institute for Biological Research “Siniša Stanković”, Bulevar Despota Stefana 142, 11000 Belgrade, Serbia University of Belgrade Belgrade Serbia

**Keywords:** Biogeography, *
Bufotes
*, Central Asia, faunistics, historical data, museum collection, new records, Reptilia, Squamata

## Abstract

Afghanistan is a herpetologically understudied country with few published papers since the end of “Afghanistan’s Golden Age” from the 1930s to the 1970s. Although a detailed checklist of the herpetofauna of the country, based on exploration of herpetodiversity using biodiversity archives, has been published recently, there still exist additional historical data that have not been considered. This is the case for a so far unknown collection of lizards from Afghanistan deposited in the herpetological collection of the Institute for Biological Research “Siniša Stanković at the University of Belgrade, Belgrade, Serbia. The material comes from field research conducted in 1972 and contains 27 specimens in seven lizard genera representing four families (Agamidae, Gekkonidae, Lacertidae, Scincidae). This historical collection was examined and basic morphometric data, field data, and photographs are provided, comparing the distributional data with published datasets. Updated species distribution maps reveal new locality or province records and an important range extension for *Eurylepis taeniolata* Blyth, 1854 which represents the northernmost record for this species in Afghanistan. In addition, one further distribution record for the *Bufotes viridis* (Laurenti, 1768) complex from the same research trip is noted.

## Introduction

A transition zone between the Palearctic and Oriental faunas, a species hotspot for some amphibians and reptiles (Hynobiidae, Agamidae, Lacertidae, Colubridae), areas that never been zoologically explored, and unknown or taxonomically unresolved species; these reasons make Afghanistan one of the most important herpetological regions of the world (Leviton and Anderson 1970, Clark 1990, Böhme and Szczerbak 1991, Wagner et al. 2016). On the other hand, 40 years of war have made the country one of the most inaccessible in the world, where current zoological or herpetological research is almost completely non-existent. This is evident in the published research related to this country (see Nahif 1986). Since the end of Afghanistan’s so-called “Golden Age” in the mid-1970s, only a few papers on its herpetofauna have been published (Böhme and Szczerbak 1991, Clark 1992, Kuch 2004, Moravec et al. 2006, Wagner et al. 2016, Jablonski and Lesko 2018). However, most of these papers are related to field research trips from the period between the 1950s and 1970s.

Wagner et al. (2016) presented a summary of most of the known material from Afghanistan in the form of an up-to-date checklist with distributional data and maps for all species based on data from biodiversity archives. According to Wagner et al. (2016), the herpetofauna of Afghanistan comprises 116 species (118 with subspecies) belonging to 58 genera and 21 families. Though the authors were consistent and examined material stored in museum and private collections, there still exist additional historical data or material not incorporated into the checklist. These include collections that were or are unknown for different reasons (forgotten personal collections, unclassified or non-catalogued collections, etc.). This is the case for the recently reported upon herpetological collection of the Institute for Biological Research “Siniša Stanković” of the University of Belgrade, Belgrade, Serbia (Džukić et al. 2017). This collection currently contains 8213 specimens originating from 23 countries. Apart from assigning collection numbers to the specimens, there have not been, until now, any systematic efforts to sort and catalogue the collection. This is why the original collection of lizards from a field trip conducted in Afghanistan during 1972 has not been previously studied. The field trip results and collected specimens were only mentioned once at the national congress of former Yugoslavia (Džukić and Vasić 1974) without any details published in the proceedings.

Because distribution data relating to the herpetofauna of Afghanistan are very important from a biogeographical point of view and comparative material from this country is rare, we have evaluated particular species and specimens stored in the Belgrade collection and compared them with information and distribution data from Wagner et al. (2016).

## Material and methods

This material comes from a field trip to Afghanistan that was conducted between 3 and 25 August 1972 by Vojislav Vasić. The specimens of herpetofauna were collected primarily during different ornithological field trips inside the country (see Vasić 1974; this work is missing in the bibliographical overview related to zoological research in Afghanistan presented by Nahif 1986). Overall, 27 specimens in seven genera are discussed. The material was originally determined to the subspecies level according to the keys of Schmidtler and Schmidtler (1969) and Leviton and Anderson (1970). Together with lizards, an additional record of the *Bufotes viridis* Laurenti, 1768 complex was mentioned (Džukić and Vasić 1974), but the voucher specimens have, unfortunately, been lost. We identified this collection to the species level and available material was coordinated with the classification of Wagner et al. (2016). Localities and dates of collection for each specimen were noted using the original labels. The material is currently in the collection of the Institute for Biological research “Siniša Stanković” preserved in 75% ethanol. Some specimens had previously been kept in the freezer or stored in formaldehyde and were recently transferred to 75% ethanol. All material is stored in labelled single-species glass jars grouped by the country and region of origin in order to facilitate their cataloguing and future work with the collection. During 2011 and 2017 the material was revised and this collection is a result of this work (Džukić et al. 2017). Whereas this collection from Afghanistan is not large and information regarding to the fauna of the country is important, all specimens were examined morphologically and photographed in detail. We examined ten basic morphological characters following Cameron et al. (2013): snout-to-vent length (SVL), body length (BL), tail width (TW), tail length (TL), jaw width (JW), jaw length (JL), fore-limb length left (FLL L), fore-limb length right (FLL R), hind-limb length left (HLL L), hind-limb length left (HLL R). Measurements were taken with a digital caliper to the nearest 0.1 mm. Morphometric data for all specimens are presented in Table 1. We have taken photos of the ventral and dorsal aspects of the specimens, as well as of details of the cloaca, the pileus, and the left and right sides of the head (see Results and Suppl. material 1). All available data relevant to each record (name of the locality, coordinates, sex, date, type of habitat) were noted and are presented. We reviewed locality data presented by Wagner et al. (2016) and made updated maps for particular species using QGIS software (2018).

**Table 1.** A basic morphometry of lizard in the collection in mm. For abbreviations of morphological features see Material and Methods. Voucher number corresponds to the jar label number in the Herpetological Collection of the Institute for Biological Research “Siniša Stanković”, University of Belgrade, Serbia. Abbreviations as given in Materials and methods.

Voucher No.	Specimen No.	Species	SVL	BL	TW	TL	JW	JL	FLL L	FLL R	HLL L	HLL R
**167**	–	* Mesalina watsonana *	45.18	36.96	5.46	61.84	7.20	11.63	8.87	8.88	14.67	14.65
**168**	–	* Eurylepis taeniolata *	78.55	66.85	6.98	110.40	8.86	14.21	8.89	8.91	12.31	12.33
**169**	–	* Eutropis dissimilis *	82.59	65.08	9.27	–	12.05	19.72	13.93	14.02	16.12	16.09
**753**	753/1	* Paralaudakia badakhshana *	77.43	61.35	10.31	101.66	15.16	20.28	10.76 + 9.56	10.64 + 9.86	17.20 + 16.54	16.20 + 16.04
**753**	753/2	* Paralaudakia badakhshana *	68.78	51.34	9.00	–	15.38	20.09	10.98 + 9.52	10.12 + 9.52	18.04 + 18.13	18.08 + 18.05
**753**	753/3	* Paralaudakia badakhshana *	73.23	57.89	10.90	125.36	16.74	19.62	12.16 + 10.51	12.19 + 10.52	17.16 + 16.99	16.81 + 17.13
**753**	753/4	* Paralaudakia badakhshana *	80.50	63.41	12.20	137.35	18.30	20.43	13.77 + 10.94	13.16 + 10.87	20.50 + 17.43	20.53 + 17.38
**779**	779/1	* Ablepharus lindbergi *	48.46	37.29	4.18	60.14	5.54	8.56	7.50	7.52	10.42	10.38
**779**	779/2	* Ablepharus lindbergi *	45.38	35.53	5.04	–	6.52	10.24	7.81	7.85	12.29	12.27
**795**	795/1	* Tenuidactylus turcmenicus *	62.30	46.93	6.96	–	12.86	17.23	8.77 + 9.88	8.62 + 9.89	14.22 + 12.67	13.80 + 12.12
**795**	795/2	* Tenuidactylus turcmenicus *	62.93	46.89	7.26	89.58	15.30	18.07	8.12 + 10.26	8.41 + 10.70	14.36 + 13.15	14.48 + 13.46
**795**	795/3	* Tenuidactylus turcmenicus *	39.99	30.63	3.86	52.00	9.02	12.15	6.31 + 7.06	6.25 + 7.07	9.31 + 8.49	9.42 + 8.48
**795**	795/4	* Tenuidactylus turcmenicus *	58.33	43.60	5.15	79.44	12.40	16.18	7.52 + 10.02	7.59 + 10.12	14.25 + 12.56	14.20 + 12.52
**795**	795/5	* Tenuidactylus turcmenicus *	64.17	47.41	6.70	76.07	13.03	16.62	7.48 + 11.06	7.46 + 10.90	15.36 + 13.30	15.19 + 13.62
**795**	795/6	* Tenuidactylus turcmenicus *	56.03	41.25	5.16	75.27	11.87	15.43	6.36 + 8.94	6.19 + 8.90	12.15 + 13.13	12.10 + 13.19
**795**	795/7	* Tenuidactylus turcmenicus *	48.05	35.38	4.63	–	10.71	14.04	6.24 + 8.23	6.34 + 8.28	12.72 + 11.21	12.82 + 11.29
**795**	795/8	* Tenuidactylus turcmenicus *	47.87	36.93	4.68	–	10.73	13.64	6.26 + 7.88	6.20 + 7.87	10.85 + 10.47	10.80 + 10.32
**795**	795/9	* Tenuidactylus turcmenicus *	64.21	47.95	5.99	90.76	12.68	16.98	8.96 + 10.84	8.86 + 10.22	16.60 + 14.08	16.25 + 14.04
**795**	795/10	* Tenuidactylus turcmenicus *	55.53	41.65	4.90	–	11.04	15.27	7.43 + 8.51	7.46 + 8.48	12.79 + 11.16	12.46 + 11.43
**887**	887/A	* Trapelus megalonyx *	68.64	50.94	9.30	75.89	17.22	19.89	9.82 + 10.31	9.87 + 12.07	15.80 + 16.91	15.24 + 15.89
**887**	887/B	* Trapelus megalonyx *	34.18	22.03	3.97	41.89	9.58	11.01	5.63 + 5.62	5.30 + 5.58	8.08 + 8.72	8.63 + 8.62
**912**	912/1	* Paralaudakia caucasia *	131.06	104.55	17.33	–	22.58	32.77	18.10 + 19.18	18.45 + 19.20	30.41 + 27.43	31.07 + 27.93
**912**	912/2	* Paralaudakia caucasia *	88.21	67.29	12.43	137.44	19.56	24.84	13.88 + 14.38	13.66 + 14.34	24.33 + 22.63	23.21 + 21.53
**912**	912/3	* Paralaudakia caucasia *	138.74	110.38	17.83	158.23	28.78	35.81	22.34 + 17.95	21.46 + 17.05	31.21 + 29.24	31.75 + 29.35
**912**	912/4	* Paralaudakia caucasia *	103.70	80.87	15.07	–	22.08	28.87	18.17 + 14.75	17.55 + 15.00	27.70 + 25.89	26.48 + 25.80
**912**	912/5	* Paralaudakia caucasia *	90.99	69.73	14.00	–	18.25	24.29	15.12 + 13.22	15.13 + 13.28	25.32 + 21.78	24.71 + 21.69
**912**	912/6	* Paralaudakia caucasia *	117.96	95.41	18.25	–	25.05	31.52	18.91 + 15.38	18.68 + 15.94	31.96 + 28.50	31.58 + 28.70

## Results and discussion

### REPTILIA

#### Agamidae

##### *Paralaudakia badakhshana* (Anderson & Leviton, 1969), Badakhshana Rock Agama

**Figure 1.**
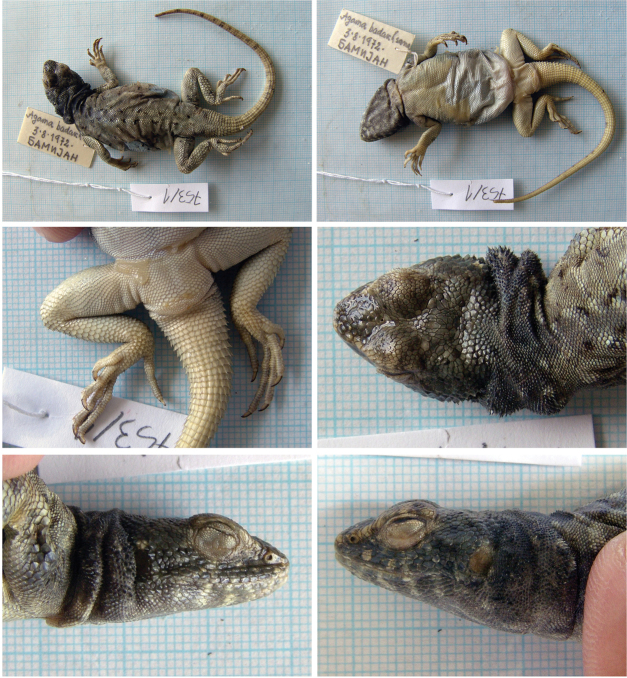
The specimen of *Paralaudakia badakhshana* no. 753/1 from Bamyan town, Bamyan. Other specimens are presented in Suppl. material 1.

Fig. 1, Suppl. material 1: Figs S1–S3

Originally identified as *Agama badakhshana*.

**Material.** Four adult specimens: 753/1 (F), 3 August 1972, Bamyan town (= Bamijan; original name on label), Bamyan, 34°48'1.65"N, 67°49'16.09"E, (desert with rocky outcrops); 753/2 (M), 753/3 (M), 753/4 (M), 4 August 1972, Azhdar-e Surkhdar (= Đavolja dolina original name on label), Bamyan, 34°49'57.68"N, 67°46'22.45"E, (desert with rocky outcrops).

**Figure 2.**
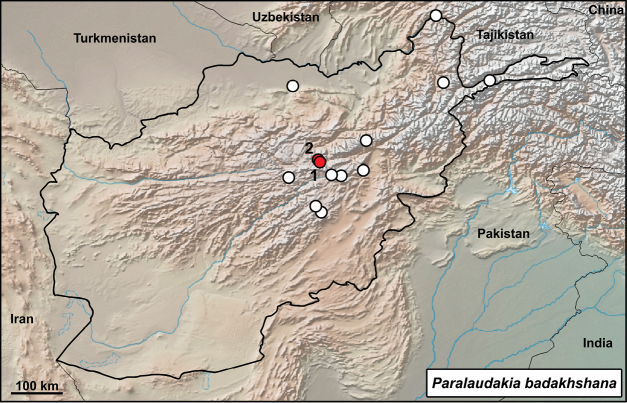
Distribution of *Paralaudakia badakhshana* in Afghanistan – white dots are from Wagner et al. (2016), red dots from this study: **1** Bamyan **2** Azhdar-e Surkhdar.

**Distribution in Afghanistan.** Mainly central parts of the Hindu Kush range, with extended records in Badakhshan (including the Wakhan corridor) and Balkh provinces. This species is currently known from the provinces of Badakhshan, Balkh, Bamyan, Ghazni, Kabul, Parwan, and Wardak (Fig. 2; Wagner et al. 2016). The record “Salang Pass, N of, road to Pulikumri [= Pol-e Khomri, Prov. Baghlan] (USNM 194973-76)” presented by Wagner et al. (2016; p. 417) is not georeferenced but will probably correspond geographically with record from “Salang Pass [Kabul Prov., 3000 m] (ZFMK 5377-81)”. Both our records are new localities for the species (Fig. 2).

##### *Paralaudakia caucasia* (Eichwald, 1831), Caucasian Agama

**Figure 3.**
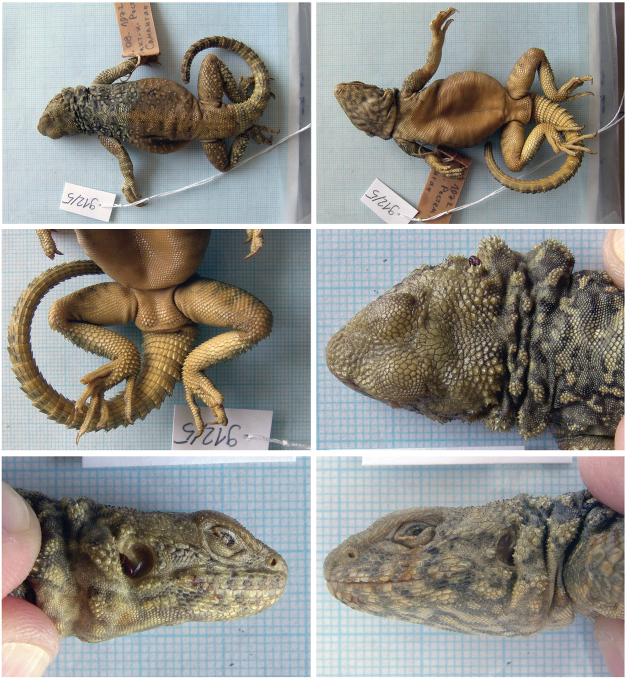
The specimen of *Paralaudakia caucasia* no. 912/5 from Takht-e Rostam, Samangan. Other specimens are presented in Suppl. material 1.

Fig. 3, Suppl. material 1: Figs S4–S8

Originally identified as *Agama caucasica*.

**Material.** Six adult specimens: 912/1 (?) and 912/2 (?), 12 August 1972, Qala-e-Naw (= Kala-I-Nav – original name on label), Badgis, 34°57'58.97"N, 63°8'41.85"E, (desert with loess profiles); 912/3 (?) and 912/4 (?), 8 August 1972, Jam (= Džam), Ghor, 34°23'45.51"N, 64°30'57.52"E, (gorge with large boulders and rocks); 912/5 (probably F) and 912/6 (M), 16 August 1972, Takht-e Rostam (=Takt - I - Rosten, Samangan), Samangan, 36°14'47.43"N, 68°1'12.29"E, (rocky desert).

**Figure 4.**
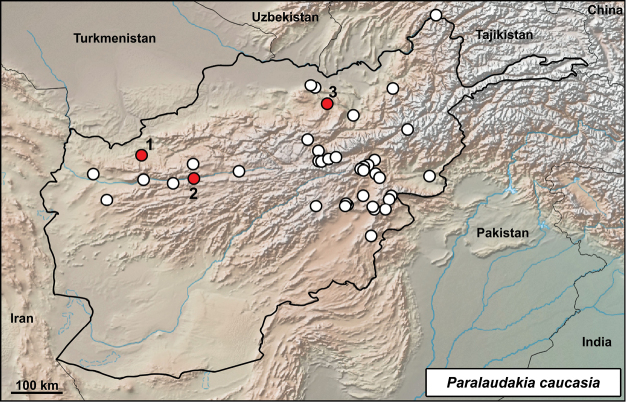
Distribution of *Paralaudakia caucasia* in Afghanistan – white dots from Wagner et al. (2016), red dots from this study: **1** Qala-e-Naw, Badgis **2** Jam, Ghor **3** Tasht-e Rostam, Samangan.

**Distribution in Afghanistan.** This species has a wide distribution range from the northwestern to the southeastern parts of the country, including northern Badakhshan. It is currently known from the provinces of Badakhshan, Badgis, Baglan, Balkh, Bamyan, Ghazni, Ghor, Herat, Kabul, Khost, Logar, Nangarhar, Paktia, Paktika, Panjshir, Takhar, and Wardak (Fig. 4; Wagner et al. 2016). The following records presented by Wagner et al. (2016; p. 472) are not georeferenced: “Bamiyan, NW of Kabul (MCZ R-97297-98)”; “40 mi S Characharan (CAS 147465)”; “Masdjed-Tchoubi (MZLU L959/3051)”; “above Pagham (Smith 1940: 384; probably BMNH 1940.3.1.18)”. Our specimens document new locality records for the species and include the first species record for Samangan Province (Fig. 4).

##### *Trapelus megalonyx* Günther, 1864, Afghan Ground Agama

**Figure 5.**
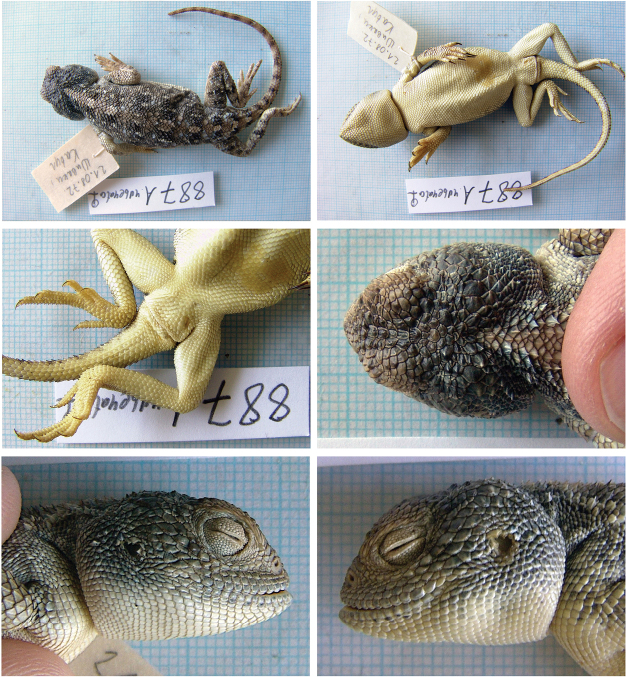
The specimen of *Trapelus megalonyx* no. 887/A from Shawarkhil, Kabul. The second specimen is presented in Suppl. material 1.

Fig. 5, Suppl. material 1: Fig. S9

Originally identified as *Agama ruderata megalonyx*.

**Material.** One adult and one juvenile specimen: 887/A (F), 21 August 1972, Shawarkhil (=Šivaki, Kabul), Kabul 34°48'3.02"N, 69°9'26.03"E, (habitat data not available); 887/B (?), 21 August 1972, Kabul – Guldara (= Kabul, Guldara), Kabul 34°45'08.89"N, 68°59'23.58"E, (rocky desert).

**Figure 6.**
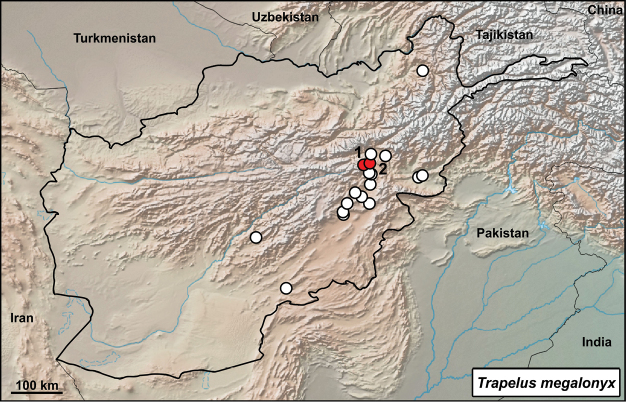
Distribution of *Trapelus megalonyx* in Afghanistan – white dots from Wagner et al. (2016), red dots from this study: **1** Kabul – Guldara, Kabul **2** Shawarkhil, Kabul.

**Distribution in Afghanistan.** This species is known mainly from south-eastern parts of the country in the provinces of Baglan, Ghazni, Kabul, Kandahar, Kapisa, Logar, Nangarhar, Uruzgan, and Wardak (Fig. 6; Wagner et al. 2016). One record from Fayzabad (Badakhshan) is not marked on the map of Wagner et al. (2016: p 481 and pl 5, p 541). Both new records document additional localities for the species (Fig. 6).

#### Gekkonidae

##### *Tenuidactylus turcmenicus* (Szczerbak, 1978), Turkmenian Thin-Toed Gecko

Fig. 7, Suppl. material 1: Figs S10–S18

Originally identified as *Cyrtodactylus fedtschenkoi*.

**Material.** Nine adult and one subadult specimens: 795/1 (F), 795/2 (M), 795/3 (?) subadult, 795/4 (F), 13 August 1972, Maymana, Faryab (= Farjab, Maymana), 35°54'54.99"N, 64°46'30.01"E, (walls of the houses in the village); 795/5 (M), 795/6 (F), 795/7 (M), 795/8 (F), 795/9 (M), 795/10 (F), 16 August 1972, Takht-e Rostam, Samangan (Takt - I - Rosten, Samangan), 36°14'47.43"N, 68°1'12.29"E, (small cave 3 km from Samangan town).

**Distribution in Afghanistan.** Mainly northern parts of the country, from approximately Bala Morgab to Kunduz (Fig. 8; Wagner et al. 2016). This species is known from the provinces of Balkh, Farah, Herat, Jowzjan, Kunduz, and Takhar. Wagner et al. (2016: 490) also mentioned the record “Seistan [Faizabad Prov.] (ZMUC R-34128)”. This record is probably incorrect as there is not a Faizabad Prov. in Afghanistan. The city Faizabad (Fayzabad) is in Badakhshan Prov. (eastern Afghanistan). Moreover, the coordinates provided by authors in the Appendix 1 are the same as for locality “Seistan [= Sistan area near Iran border]” on p 556 (western Afghanistan). Its potential distribution in Badakhshan needs further clarification. On the other hand, Wagner et al. (2016: 490) presented the record “Kouh-Akhour near Farah (NMW 15879)” which is not shown on their map, but represents the southern- and easternmost locality of the species in Afghanistan (see map in Fig. 8 and compare it with the species map in Wagner et al. 2016: pl 6, p 542). This range extension needs further clarification. Both records reported here represent new locality records for the species and first records for the provinces of Faryab and Samangan (Fig. 8).

**Figure 7.**
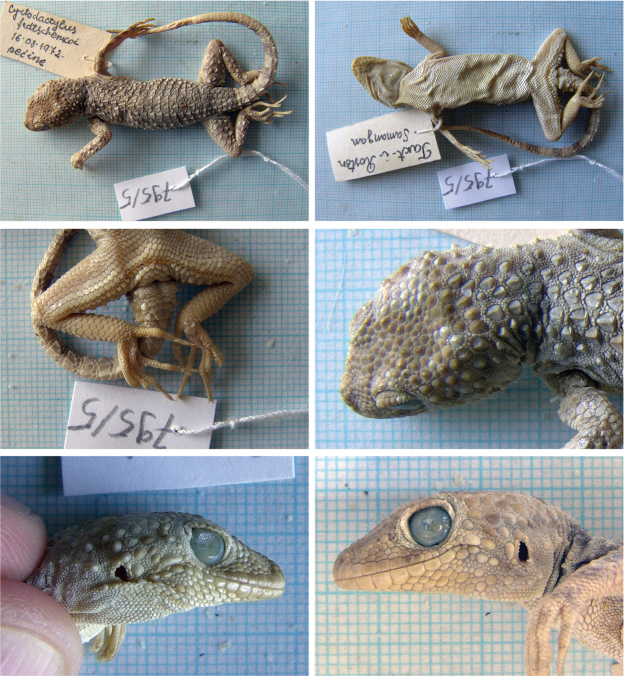
The specimen of *Tenuidactylus turcmenicus* no. 795/5 from Takht-e Rostam, Samangan. Other specimens are presented in Suppl. material 1.

**Figure 8.**
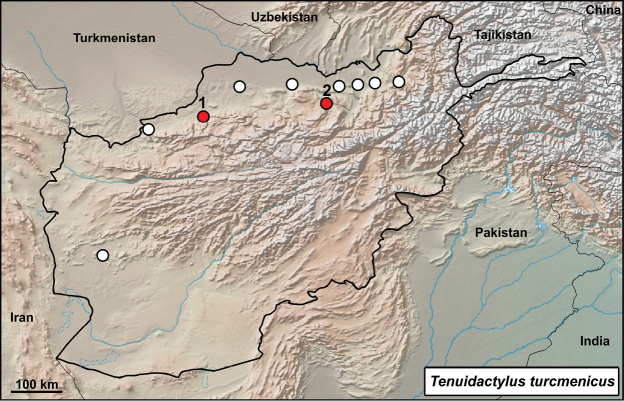
Distribution of *Tenuidactylus turcmenicus* in Afghanistan – white dots from Wagner et al. (2016), red dots from this study: **1** Maymana, Faryab **2** Takht-e Rostam, Samangan.

#### Lacertidae

##### *Mesalina watsonana* (Stoliczka, 1872), Persian Long-Tailed Desert Lizard

Fig. 9

Originally identified as *Eremias guttulata*.

**Material.** One adult specimen: 167 (M), 21 August 1972, Kabul – Guldara, Kabul (= Kabul, Guldara), 34°45'08.89"N, 68°59'23.58"E, (rocky desert).

**Distribution in Afghanistan.** A common species with a number of records mainly from southern Afghanistan below the Hindu Kush Range. It is currently known from the following provinces: Badakhshan, Farah, Ghazni, Ghor, Helmand, Herat, Kabul, Kandahar, Khost, Logar, Nangarhar, Paktia, Paktika, Parwan, Uruzgan, Wardak, and Zabul (Wagner et al. 2016 and Fig. 10). Two localities mentioned by Wagner et al. (2016: 498): “40 km NE of Kandhar, on Tarnak River (CAS 90757-60” and “Mil-Karez, Pol-Mil (MZLU L958/3230)” are not presented with coordinates. Therefore, they are not included in the map (Fig. 10). Guldara is an additional record for the species, whereas this lizard is known from many of records in Kabul Province (Fig. 10).

**Figure 9.**
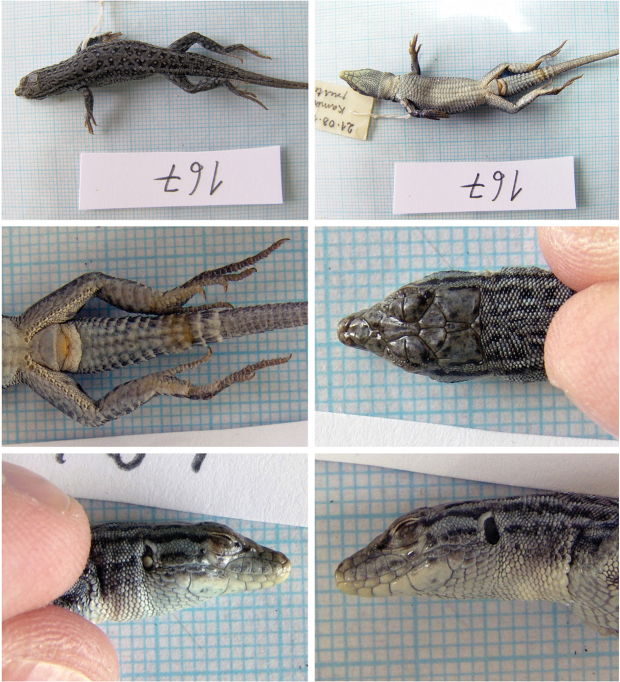
The specimen of *Mesalina watsonana* no. 167 from Kabul – Guldara, Kabul.

**Figure 10.**
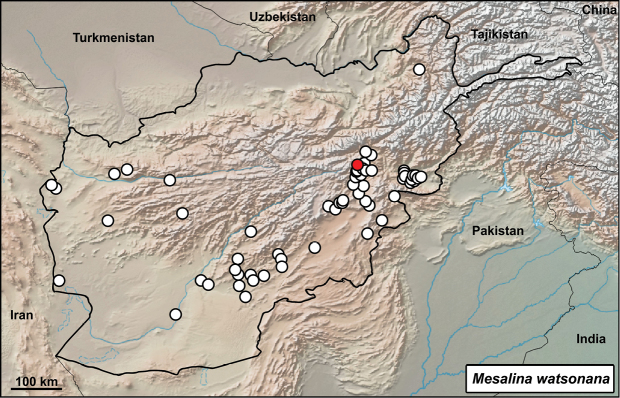
Distribution of *Mesalina watsonana* in Afghanistan – white dots from Wagner et al. (2016), red dot from this study: Kabul – Guldara, Kabul.

#### Scincidae

##### *Ablepharus lindbergi* Wettstein, 1960, Lindberg’s Snake-Eyed Skink

**Figure 11.**
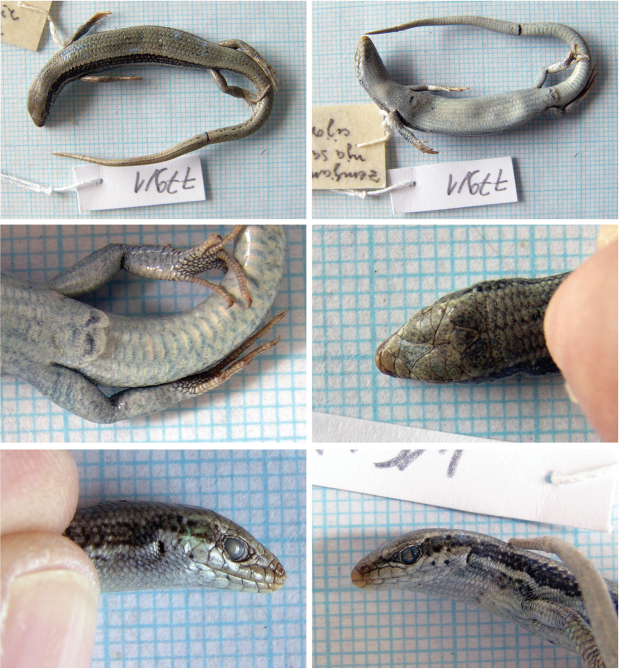
The specimen of *Ablepharus lindbergi* no. 779/1 from Band-e Amir, Bamyan. The second specimen is presented in Suppl. material 1.

Fig. 11, Suppl. material 1: Fig. S19

Originally identified as *Ablepharus bivittatus lindbergi*.

**Material.** Two adult specimens: 779/1 (?), 4 August 1972; 779/2 (?), 5 August 1972, Band-e Amir, (= Band I Amir), Bamyan, 34°50'1.51"N, 67°12'58.35"E, (arid soil desert with vegetation).

**Figure 12.**
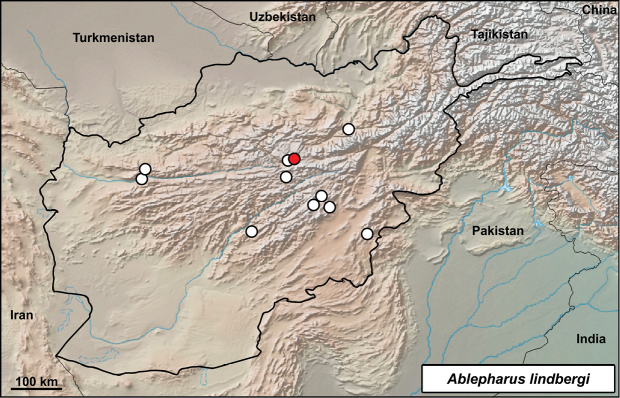
Distribution of *Ablepharus lindbergi* in Afghanistan – white dots from Wagner et al. (2016), red dot from this study: Band-e Amir, Bamyan.

**Distribution in Afghanistan.** Scattered localities in the western and central Hindu Kush and Shinkay Hills. This species is currently known from the provinces of Baghlan, Bamyan, Ghazni, Herat, Paktika and Uruzgan (Wagner et al. 2016 and Fig. 12). The following localities presented by Wagner et al. (2016; p. 499) were not georeferenced by those authors and they are not shown on the map (Fig. 12): “Kotal-e-sh-tu [Maidan Prov., western Behsud, 2000 m] (ZFMK 8664)”; “Masdjed, Tohoubi (MZLU L959/3044)”; Tshomay [Maidan Prov., western Behsud, 2000 m] (ZFMK 8663)”. The locality presented here is a new record although this lizard was previously known from this region and from the province (Fig. 12).

##### *Eurylepis taeniolata* Blyth, 1854, Yellow-bellied Mole Skink

Fig. 13

Originally identified as *Eumeces taeniolatus*.

**Material.** One adult specimen: 168 (probably M), 16 August 1972, Takht-e Rostam (= Takt - I – Rosten, Samangam), Samangan, 36°14'47.43"N, 68°1'12.29"E, (rocky desert).

**Distribution in Afghanistan.** The species is known from three localities in southern and south-eastern parts of the country, and from one locality in the northwestern part of the country (provinces of Badghis, Kandahar, Khost, and Nangarhar; Wagner et al. 2016 and Fig. 14). One locality (“Tajan River” probably from Herat province) originating from Leviton and Anderson (1970) and given also by Wagner et al. (2016), is not georeferenced and is not included in our map (Fig. 14). The locality presented here is a new record for the species and the first record for Samangan Province in northern Afghanistan. It is also the northernmost species record for Afghanistan, located more than 300 airline km from the Nangarhar record (Somarkhel) and more than 400 km from the Badghis record (Bala Murghab, Fig. 14; see also Wagner et al. 2016).

**Figure 13.**
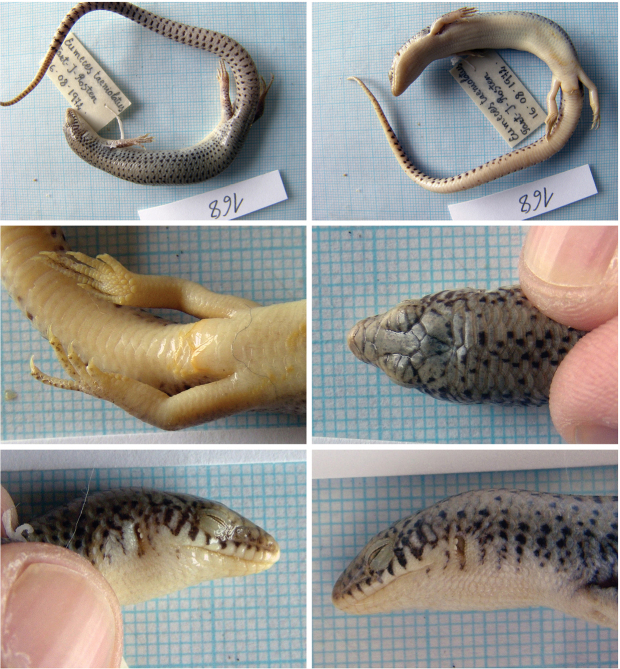
The specimen of *Eurylepis taeniolata* no. 168 from Takht-e Rostam, Samangan.

**Figure 14.**
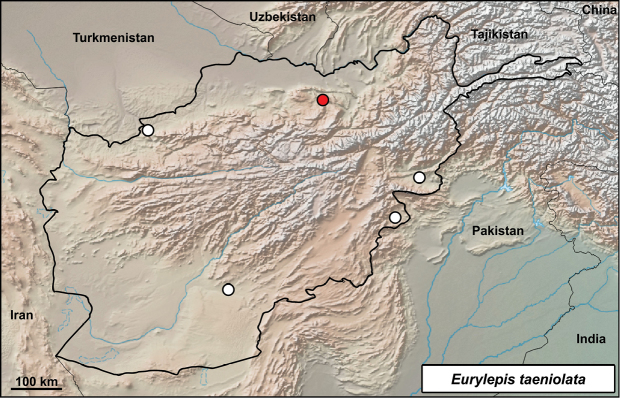
Distribution of *Eurylepis taeniolata* in Afghanistan – white dots from Wagner et al. (2016), red dot from this study: Takht-e Rostam, Samangan.

##### *Eutropis dissimilis* (Hallowell, 1857), Striped Grass Skink

Fig. 15

Originally identified as *Mabuya dissimilis*.

**Material.** One adult specimen: 169 (probably M), 25 August 1972, Jalalabad – Hadda (= Hada, Džalalabad), Nangarhar, 34°21'54.86"N, 70°28'34.37"E, (grassy patch in the desert).

**Distribution in Afghanistan.** The species is known only from three localities in the southeastern part of the country in Nangarhar Province (Wagner et al. 2016 and Fig. 16). Our locality is a new locality record for the species, although it is known from the vicinity of Jalalabad and in Nangarhar Province (Fig. 16).

**Figure 15.**
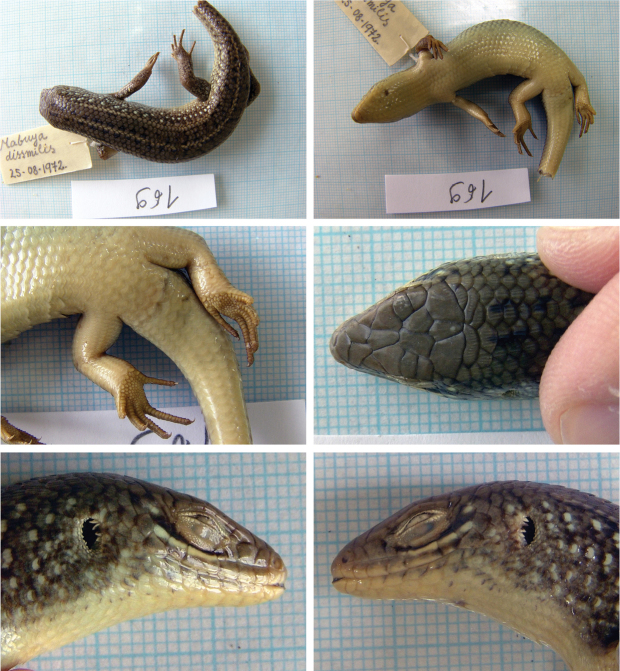
The specimen of *Eutropis dissimilis* no. 169 from Jalalabad – Hadda, Nangarhar.

**Figure 16.**
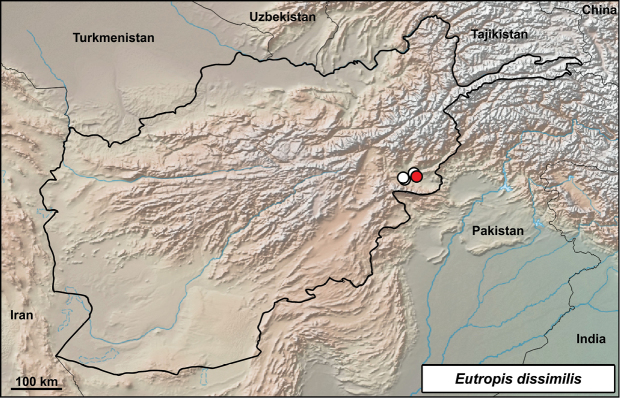
Distribution of *Eutropis dissimilis* in Afghanistan – white dots from Wagner et al. (2016), red dot from this study: Jalalabad – Hadda, Nangarhar.

### AMPHIBIA

#### Bufonidae

##### *Bufotes viridis* (Laurenti, 1768) complex, Green Toad

Fig. 17

Originally identified as: *Bufo raddei*.

**Material.** Two voucher specimens that are currently not found in the museum collection were collected on 3 August 1972 in the vicinity of Bamyan town (= Bamijan), 34°48'1.65"N, 67°49'16.09"E, (irrigation canals near the town). The specimens were identified as *Bufo raddei* based on the morphometric characters, according to Schmidtler and Schmidtler (1969).

**Figure 17.**
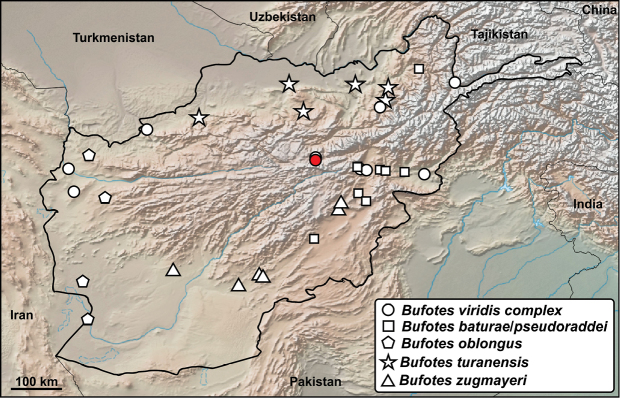
Distribution of all species forming *Bufotes viridis* complex in Afghanistan – white symbols from Wagner et al. (2016), red dot from this study: vicinity of Bamyan town, Bamyan.

**Distribution in Afghanistan.** According to Wagner et al. (2016), in Afghanistan this species complex comprises four species (*B. oblongus*, *B. pseudoraddei*, *B. turanensis*, *B. zugmayeri*) that are recorded through the whole of the country except the central Hindu Kush Range (Fig. 17). These toads are known from the provinces of Badakhshan, Badghis, Baglan, Balkh, Bamyan, Farah, Faryab, Ghazni, Helmand, Herat, Kabul, Kandahar, Kunduz, Logar, Nangarhar, Paktia, Samangan, Takhar, Wardak, and Zabul (Wagner et al. 2016 and Fig. 17). One locality presented by Wagner et al. (2016: 463) for *B. pseudoraddei*, “Culangor [Logar Prov.] (USNM 194595-97)”, lacks georeferenced data and is not included on the map. For additional information and unclear localities see remarks (p 462) in Wagner et al. (2016). The specimens noted here confirm a record treated as incertae sedis within the *Bufotes viridis* complex for Bamyan (Wagner et al. 2016). The record from Bamyan probably belongs to *B. baturae* (Stöck, Schmid, Steinlein, and Grosse 1999). Whereas Wagner et al. (2016) mentioned this taxon as a subspecies of *B. pseudorradei* (Mertens, 1971) and Frost et al. (2019) presented both as independent species occurring in Afghanistan (Stöck et al. 2006, Betto-Colliard et al. 2015), we present records of these toads under *B. baturae*/*pseudoraddei* (Fig. 17). The distribution and taxonomy of these toads in Afghanistan needs further research.

## Supplementary materials

Supplementary material 1 Figures S1–S19 Data type: Adobe Acrobat Document (.pdf) Explanation note: Additional specimens of lizards collected in Afghanistan and their localities in the country from the herpetological collection of the Institute for Biological research “Siniša Stanković”, University of Belgrade, Belgrade, Serbia. https://binary.pensoft.net/file/300330This dataset is made available under the Open Database License (http://opendatacommons.org/licenses/odbl/1.0/). The Open Database License (ODbL) is a license agreement intended to allow users to freely share, modify, and use this Dataset while maintaining this same freedom for others, provided that the original source and author(s) are credited.Daniel Jablonski, Aleksandar Urošević, Marko Andjelković, Georg Džukić
