# Supplementary material for: An unknown collection of lizards from Afghanistan
Source: Zookeys. 2019 May 9;843:129–47. doi: 10.3897/zookeys.843.29420 (PMC6522455; doi:10.3897/zookeys.843.29420)

An unknown collection of lizards from Afghanistan

Daniel Jablonski, Aleksandar Urosević, Marko Andjelković, Georg Džukić

SUPPLEMENTARY FILE 1: Figures S1-S19.

Additional specimens of lizards collected in Afghanistan and their localities in the country from the herpetological collection of the Institute for Biological research "Siniša Stanković", University of Belgrade, Belgrade, Serbia.

Figure S1. The specimen of *Paralaudakia badakhshana* no. 753/2 from Azhdar-e Surkhdar, Bamyan.

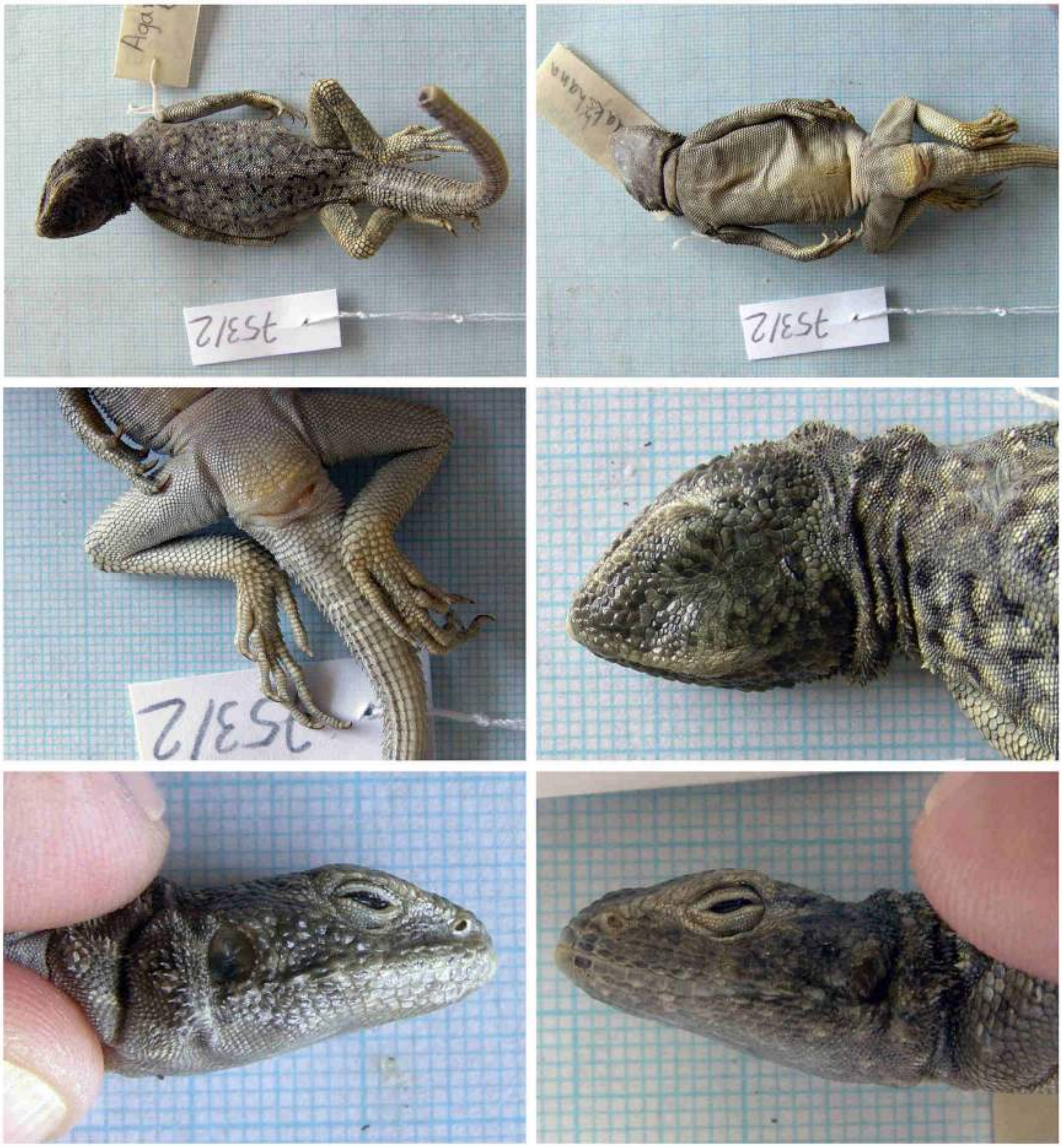

Figure S2. The specimen of *Paralaudakia badakhshana* no. 753/3 from Azhdar-e Surkhdar, Bamyan.

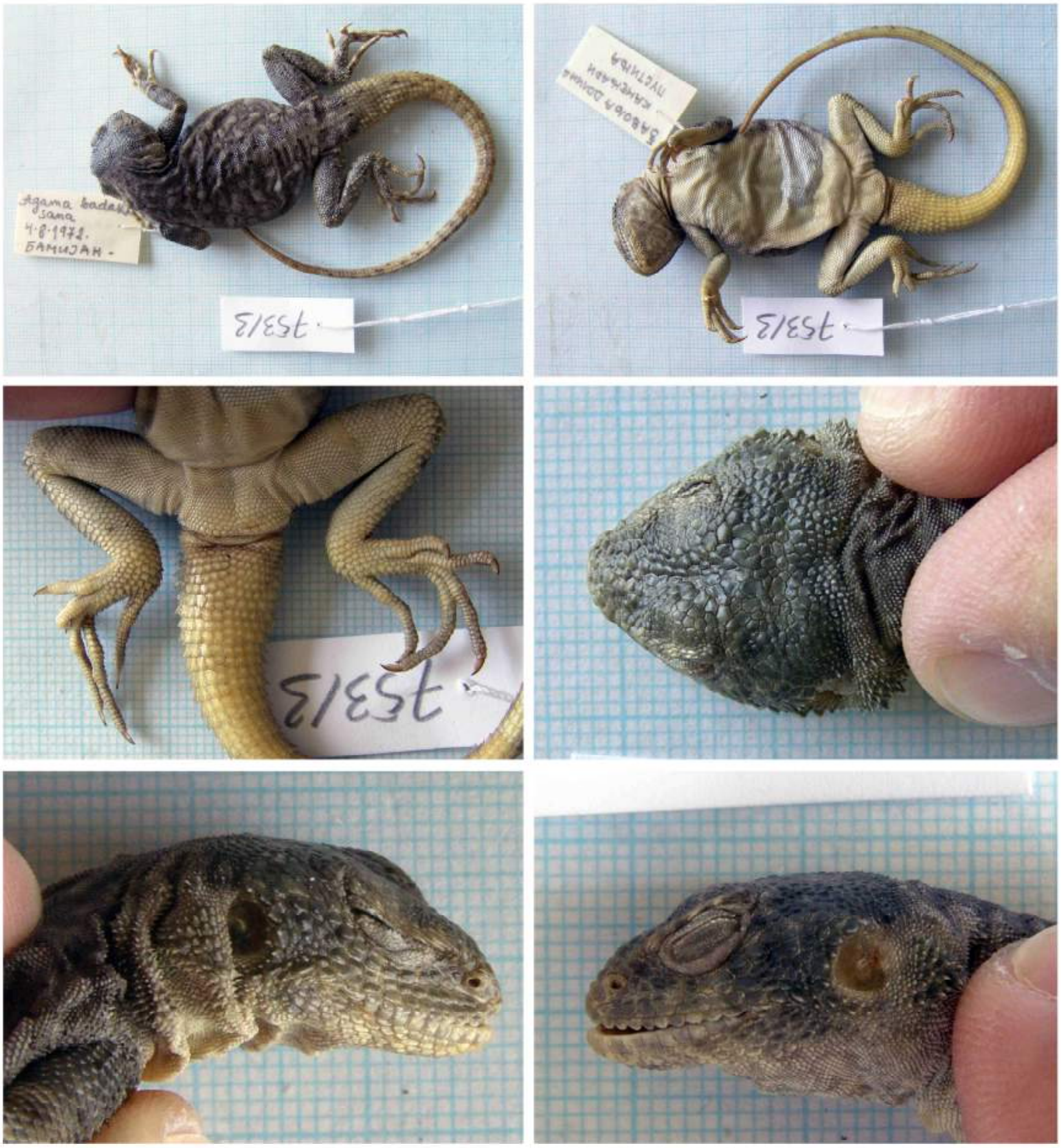

Figure S3. The specimen of *Paralaudakia badakhshana* no. 753/4 from Azhdar-e Surkhdar, Bamyan.

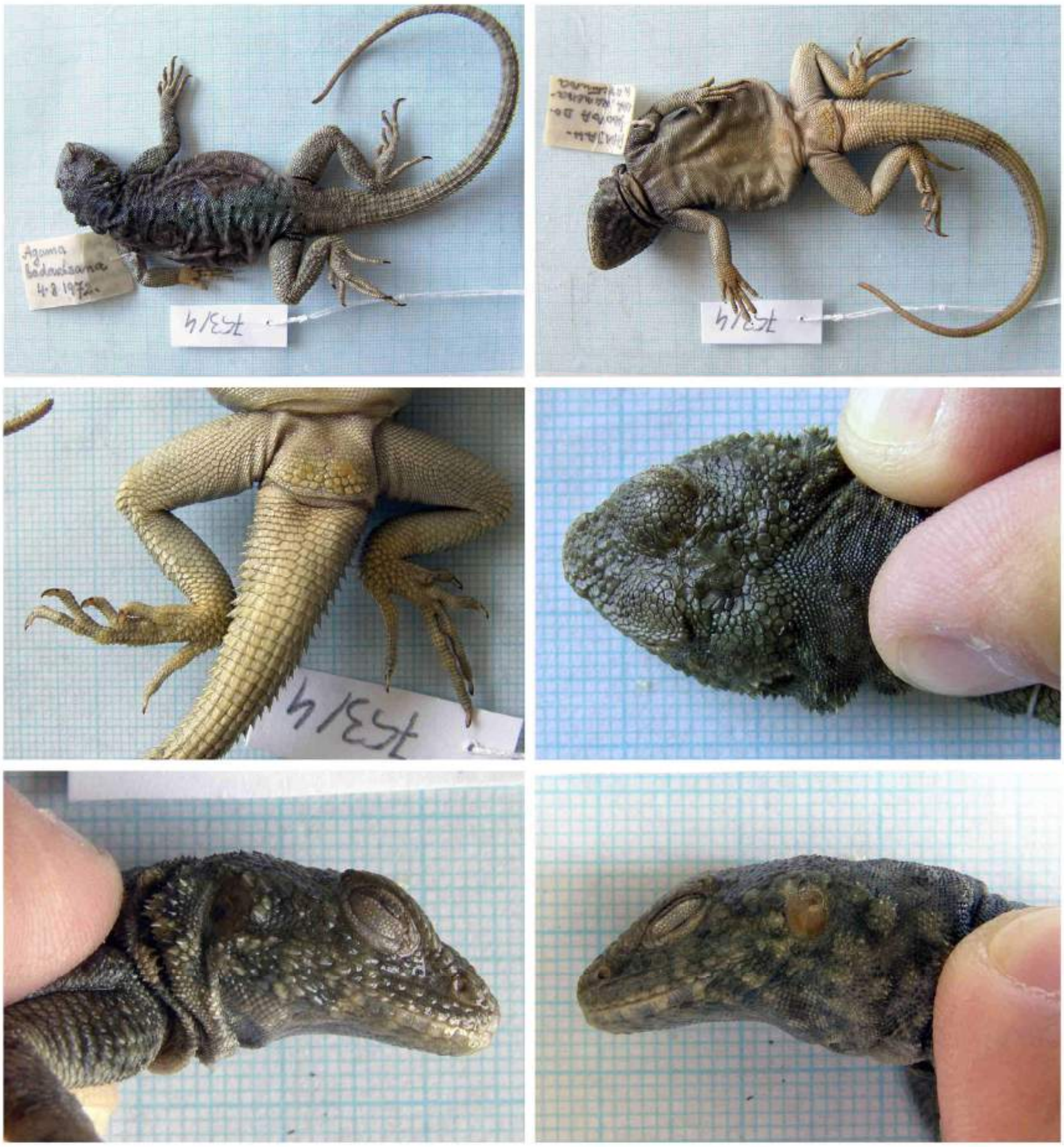

Figure S4. The specimen of *Paralaudakia caucasia* no. 912/1 from Qala-e-Naw, Badgis

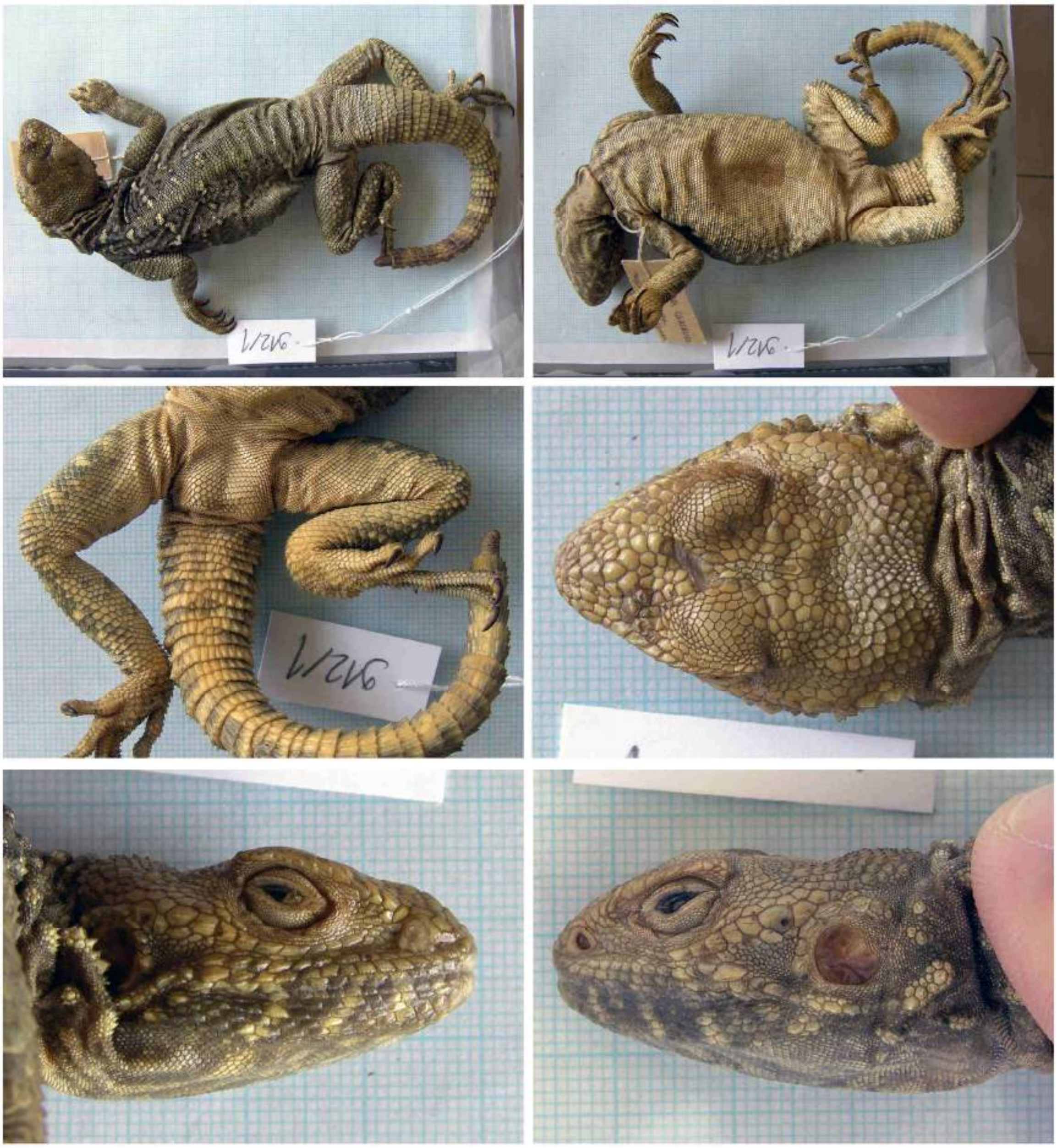

Figure S5. The specimen of *Paralaudakia caucasia* no. 912/2 from Qala-e-Naw, Badgis.

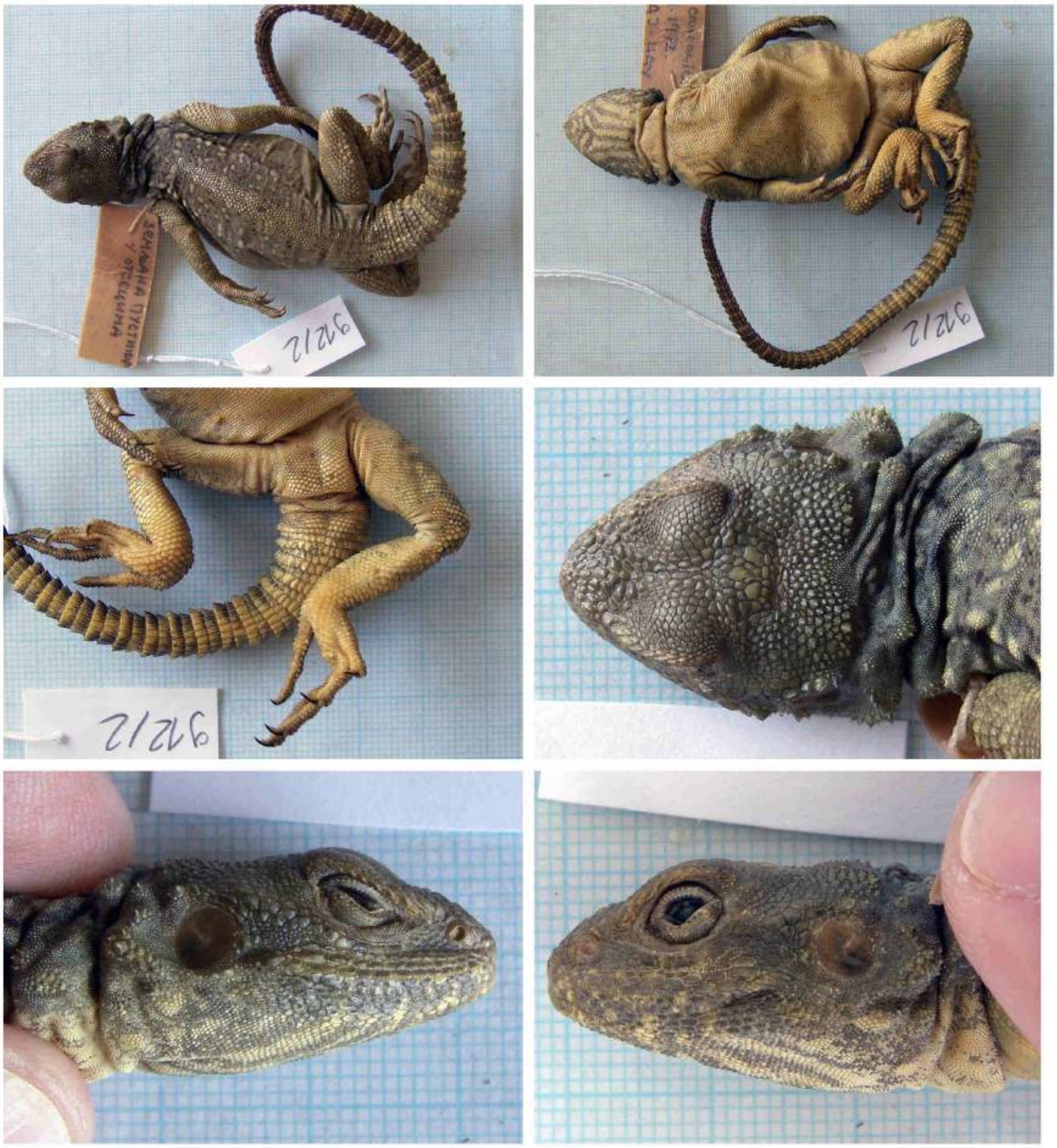

Figure S6. The specimen of *Paralaudakia caucasia* no. 912/3 from Jam, Ghor.

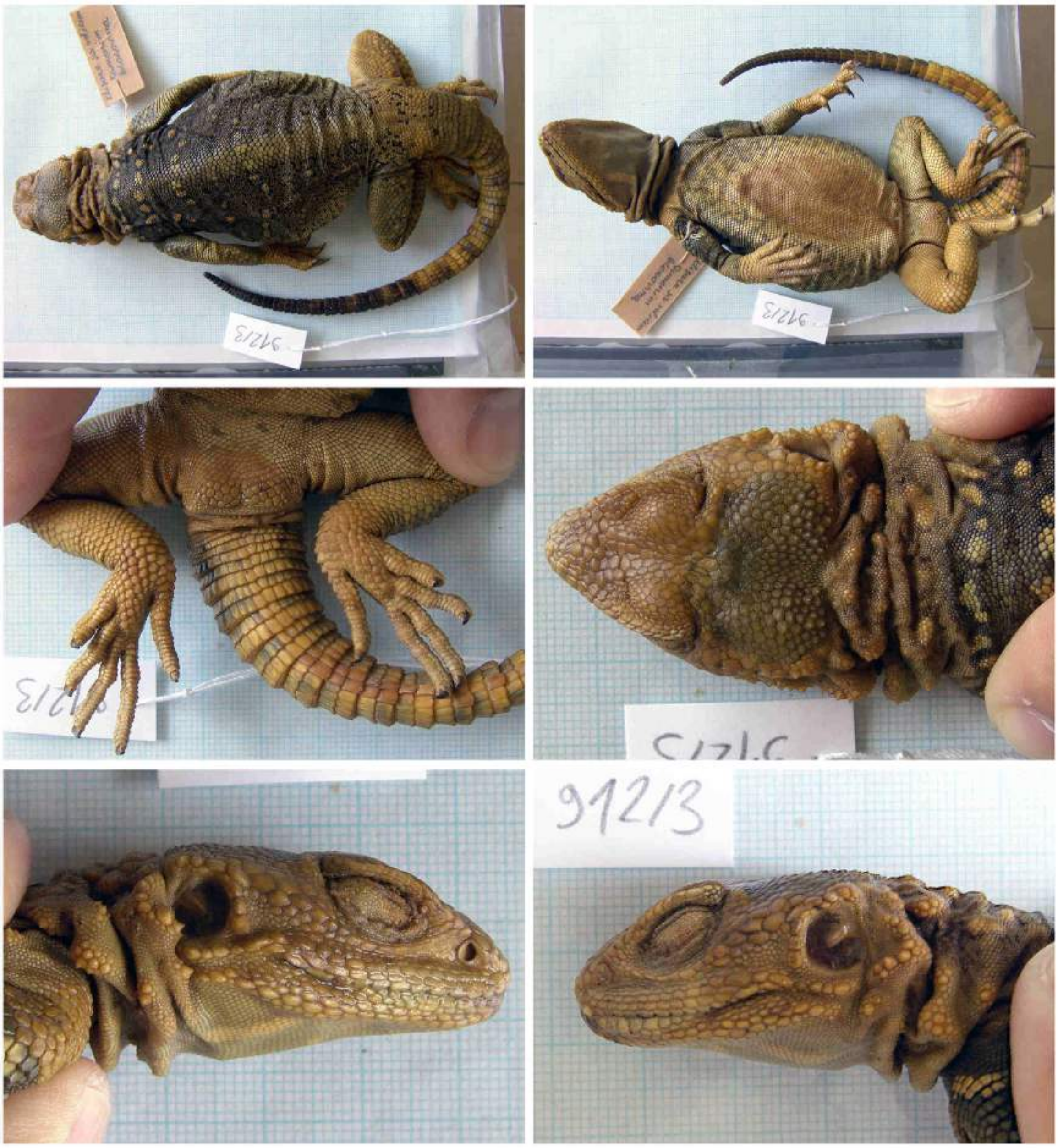

Figure S7. The specimen of *Paralaudakia caucasia* no. 912/4 from Jam, Ghor.

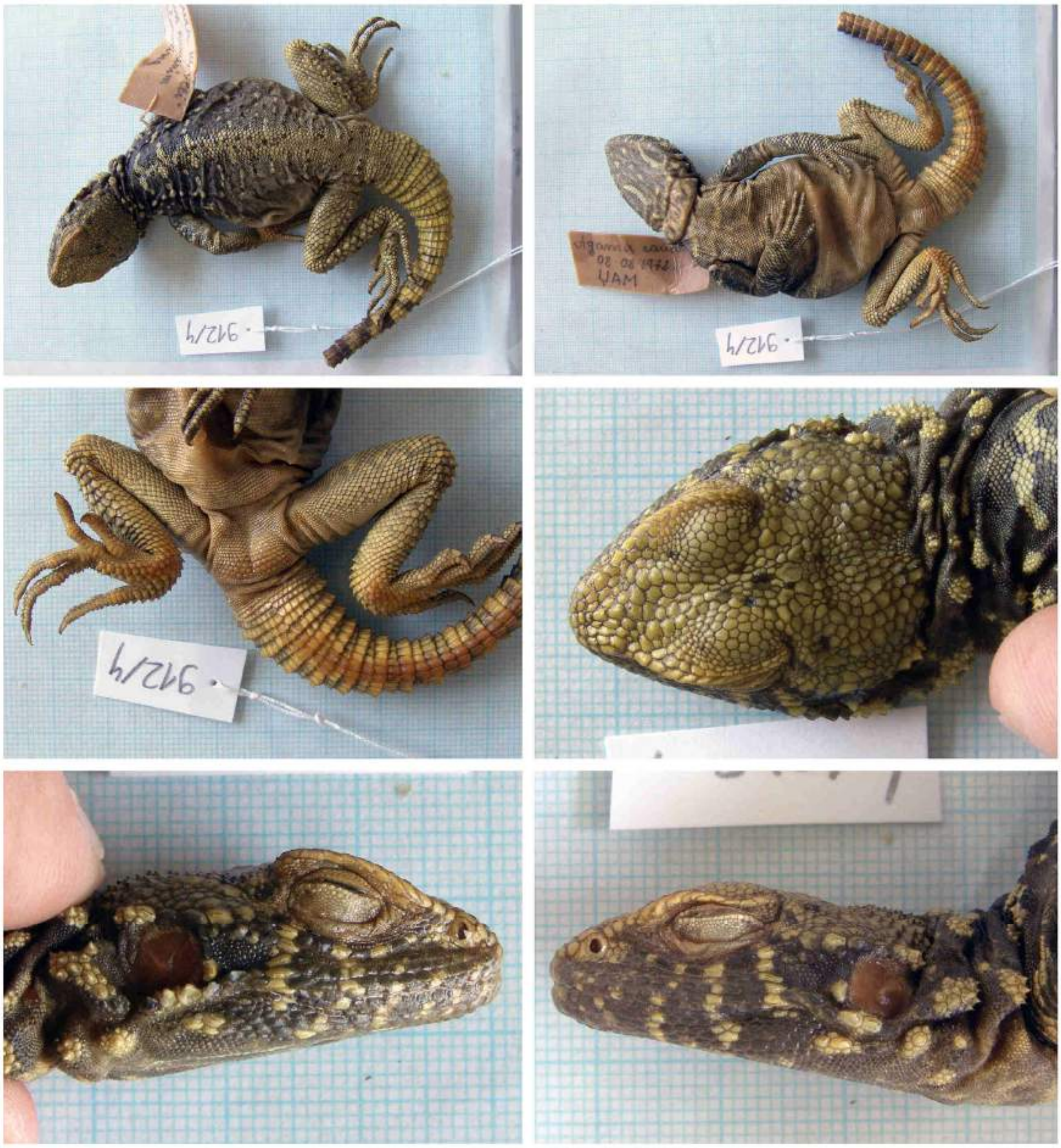

Figure S8. The specimen of *Paralaudakia caucasia* no. 912/6 from Takht-e Rostam, Samangan.

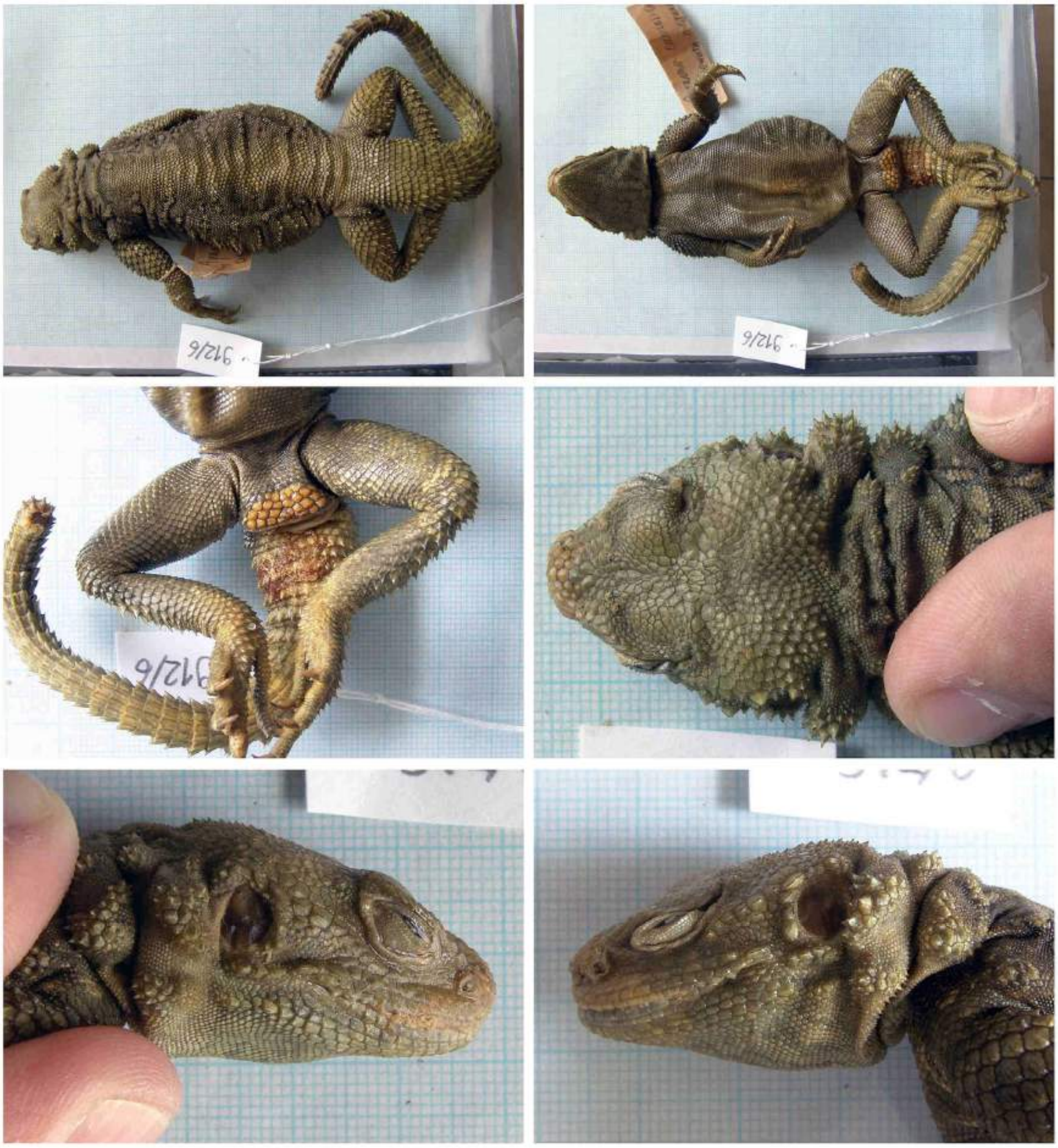

Figure S9. The specimen of *Trapelus megalonyx* no. 887/B from Kabul – Guldara, Kabul.

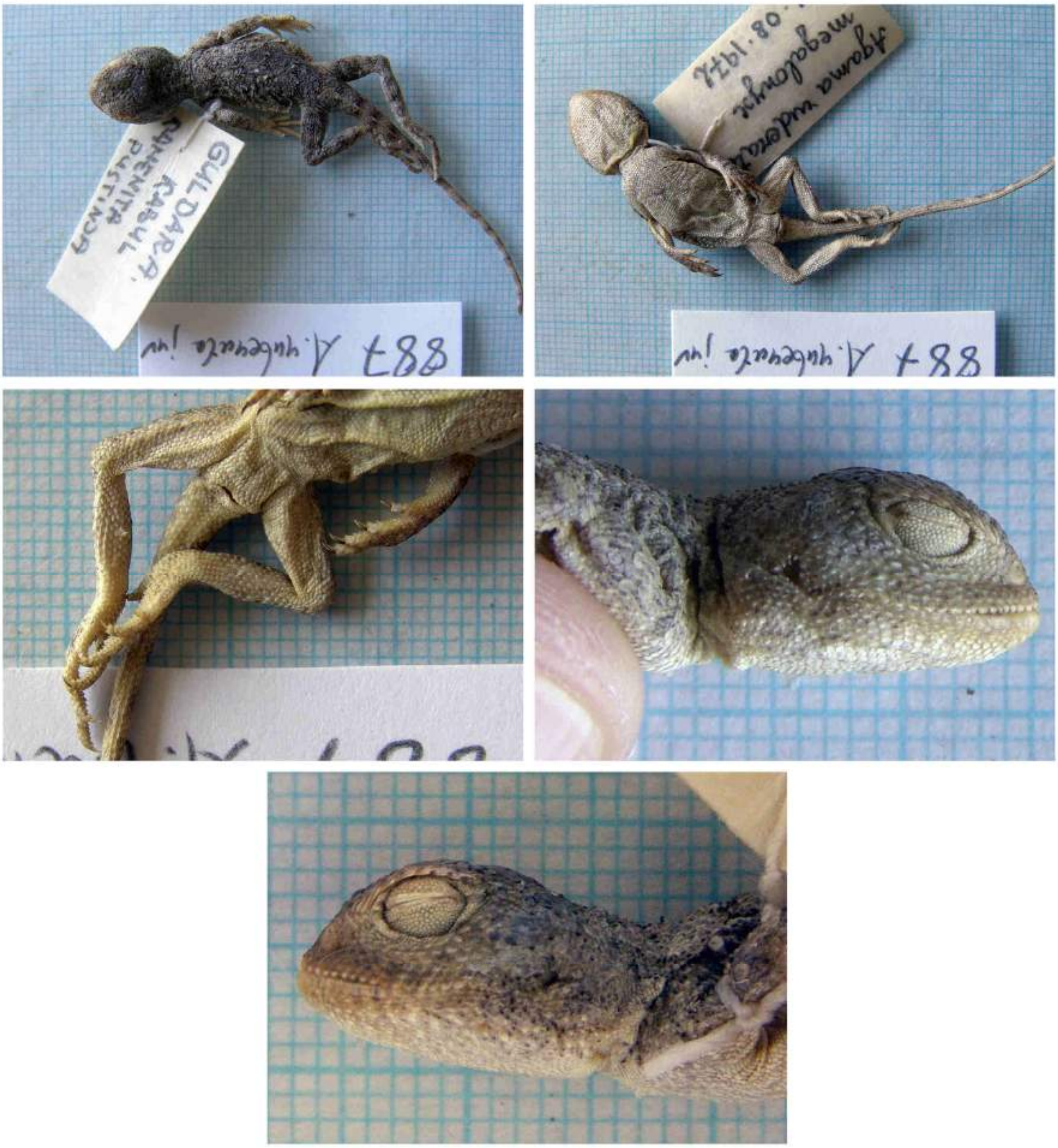

Figure S10. The specimen of *Tenuidactylus turcmenicus* no. 795/1 from Maymana, Faryab.

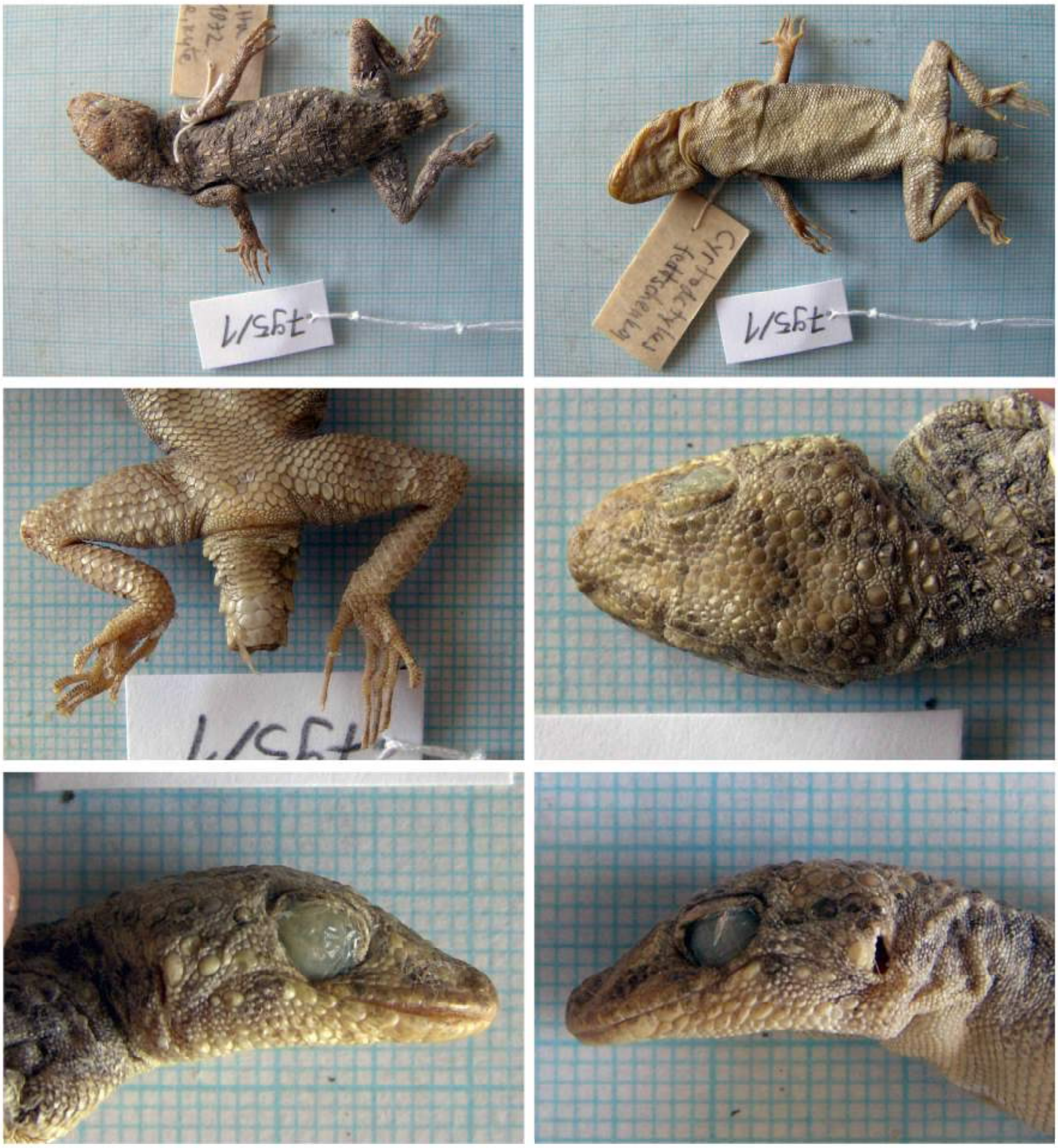

Figure S11. The specimen of *Tenuidactylus turcmenicus* no. 795/2 from Maymana, Faryab.

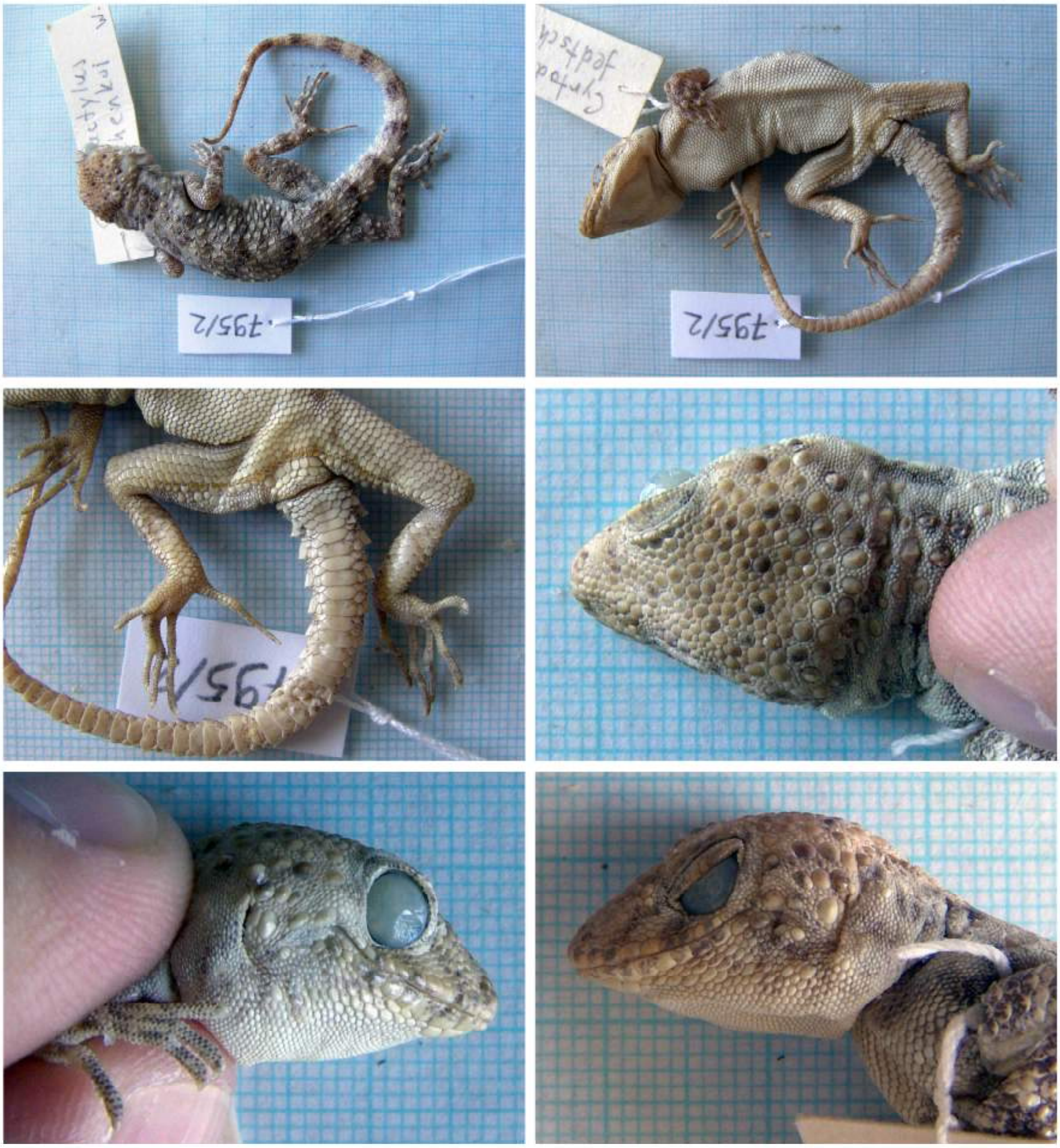

Figure S12. The specimen of *Tenuidactylus turcmenicus* no. 795/3 from Maymana, Faryab.

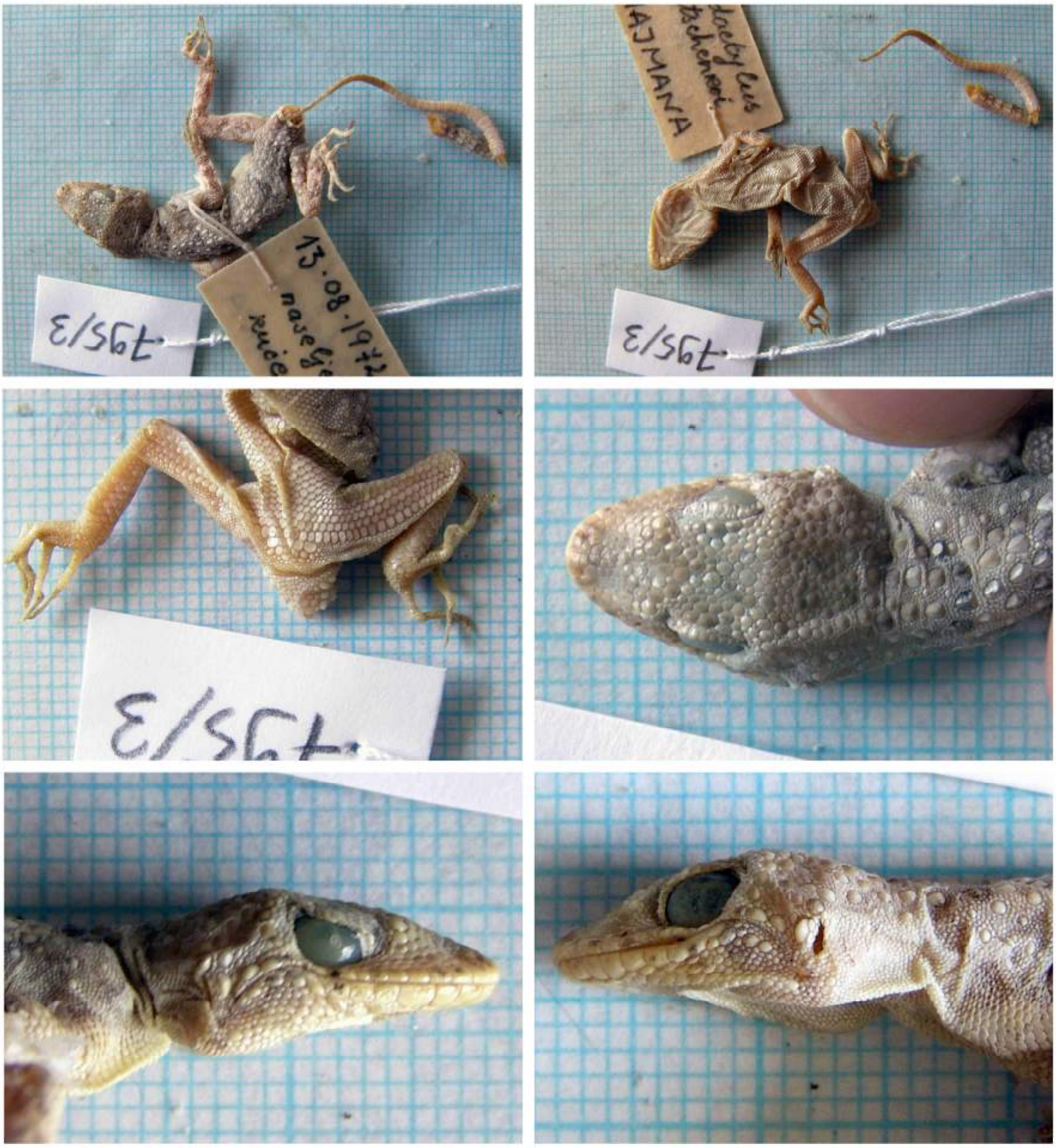

Figure S13. The specimen of *Tenuidactylus turcmenicus* no. 795/4 from Maymana, Faryab.

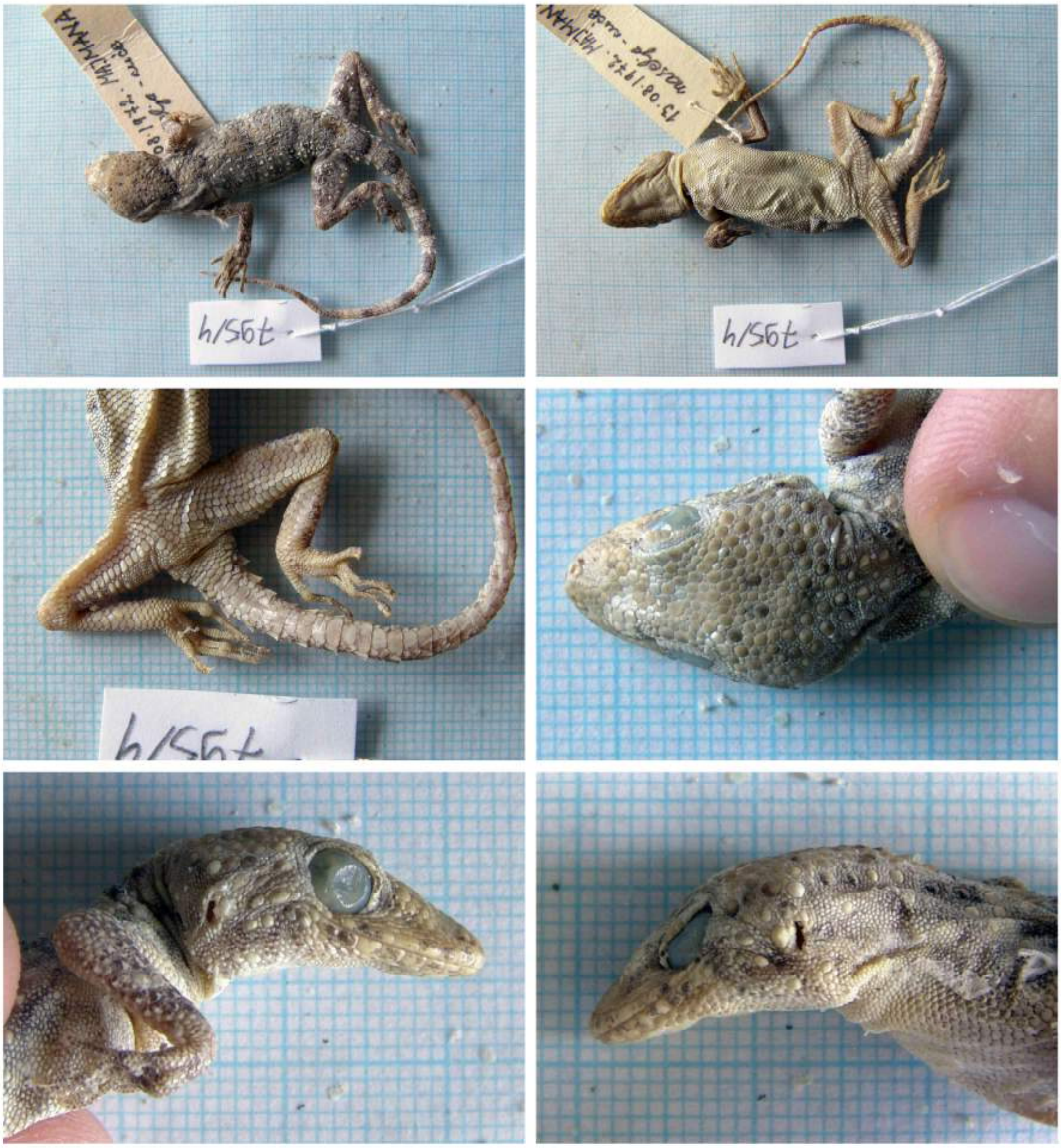

Figure S14. The specimen of *Tenuidactylus turcmenicus* no. 795/6 from Takht-e Rostam, Samangan.

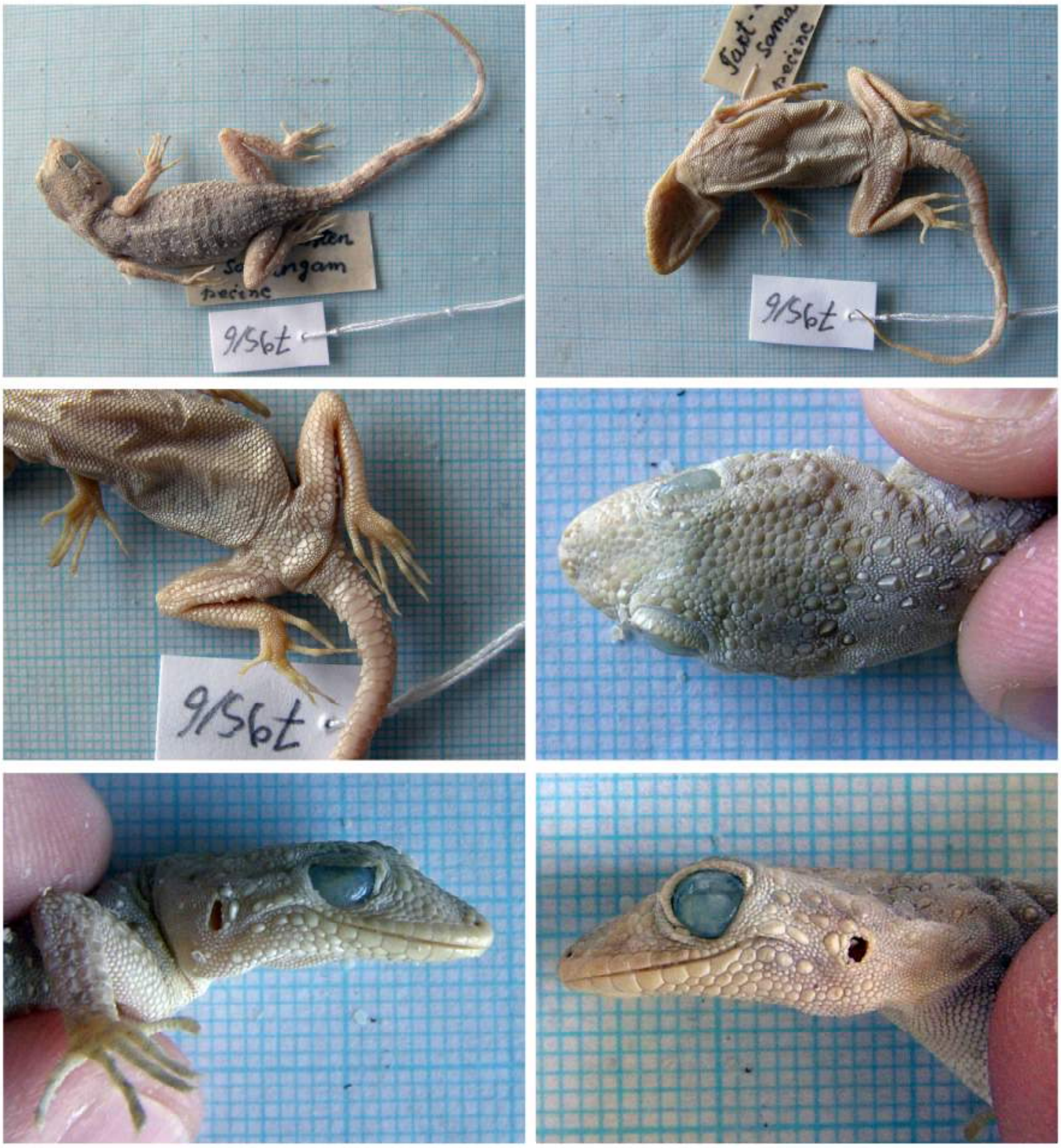

Figure S15. The specimen of *Tenuidactylus turcmenicus* no. 795/7 from Takht-e Rostam, Samangan.

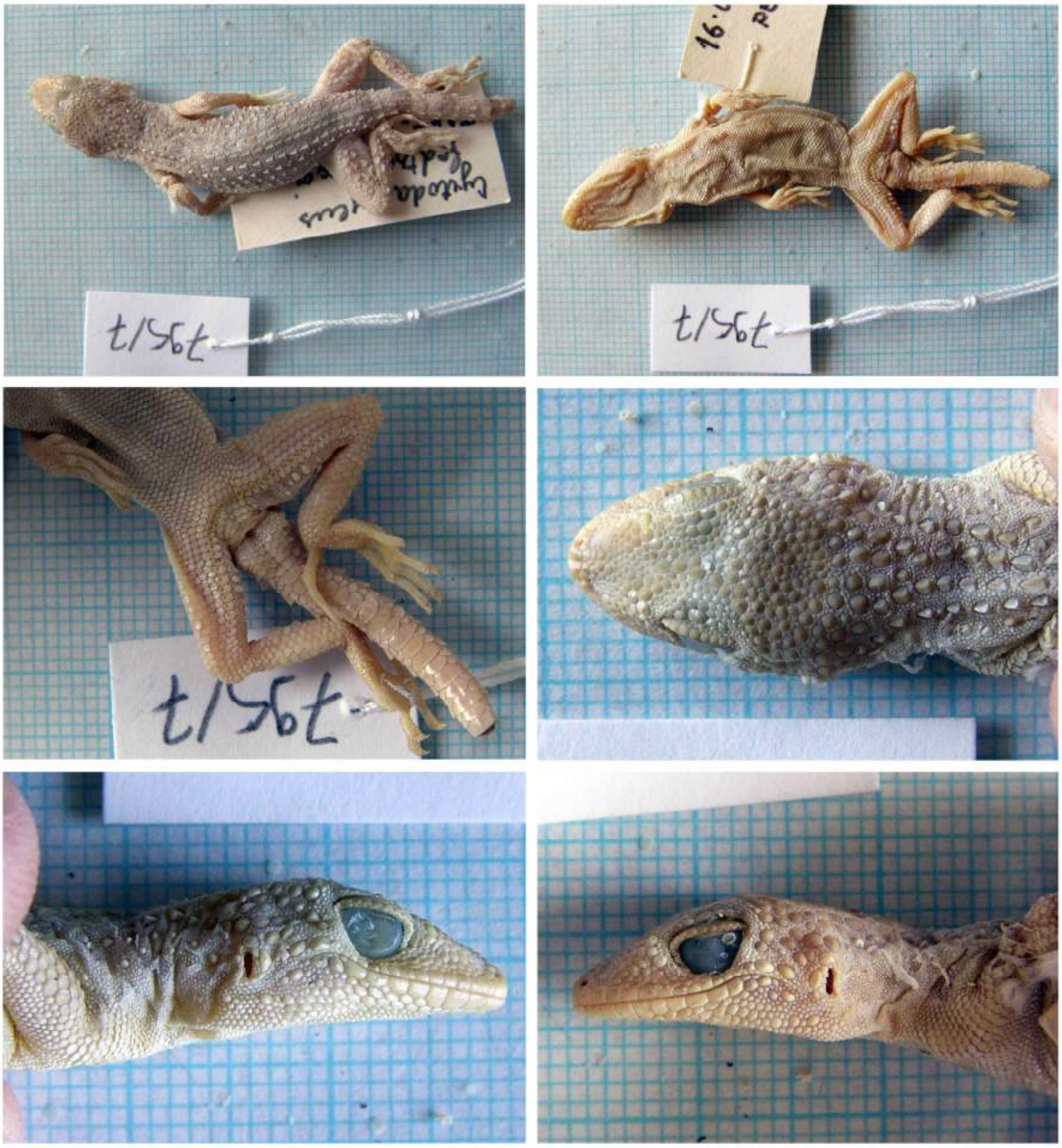

Figure S16. The specimen of *Tenuidactylus turcmenicus* no. 795/8 from Takht-e Rostam, Samangan.

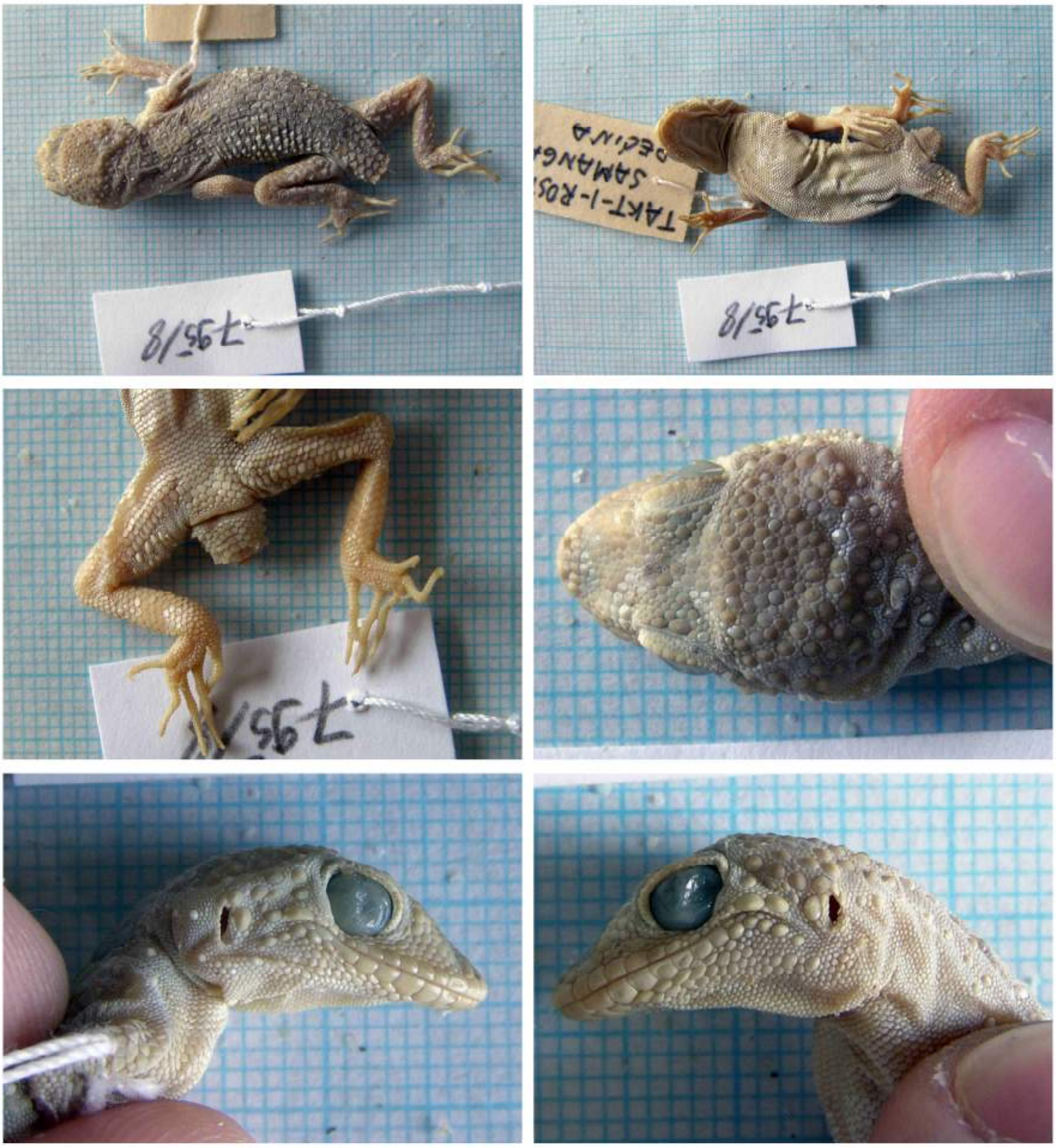

Figure S17. The specimen of *Tenuidactylus turcmenicus* no. 795/9 from Takht-e Rostam, Samangan.

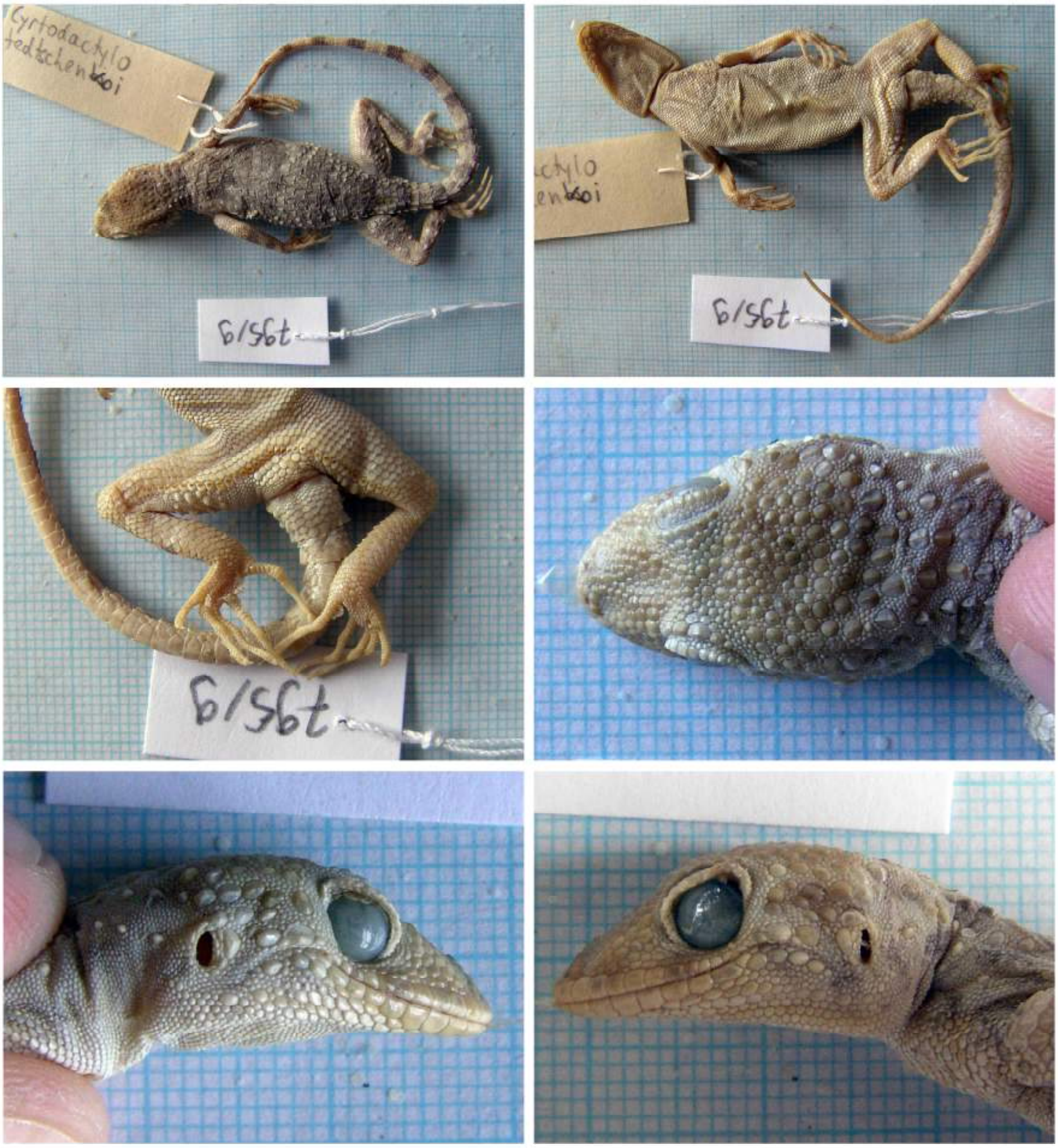

Figure S18. The specimen of *Tenuidactylus turcmenicus* no. 795/10 from Takht-e Rostam, Samangan.

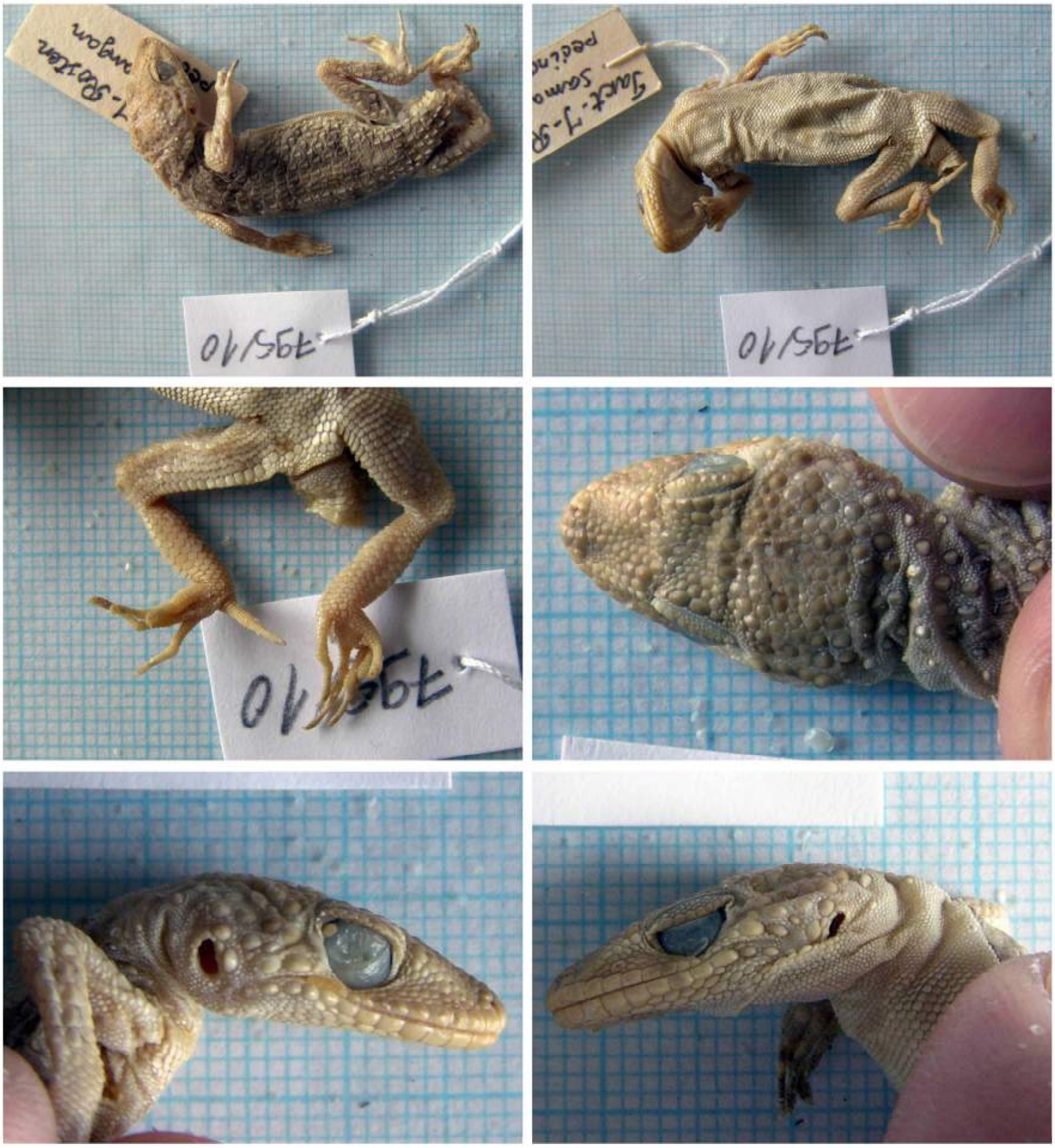

Figure S19. The specimen of *Ablepharus lindbergi* no. 779/2 from Band-e Amir, Bamyan.

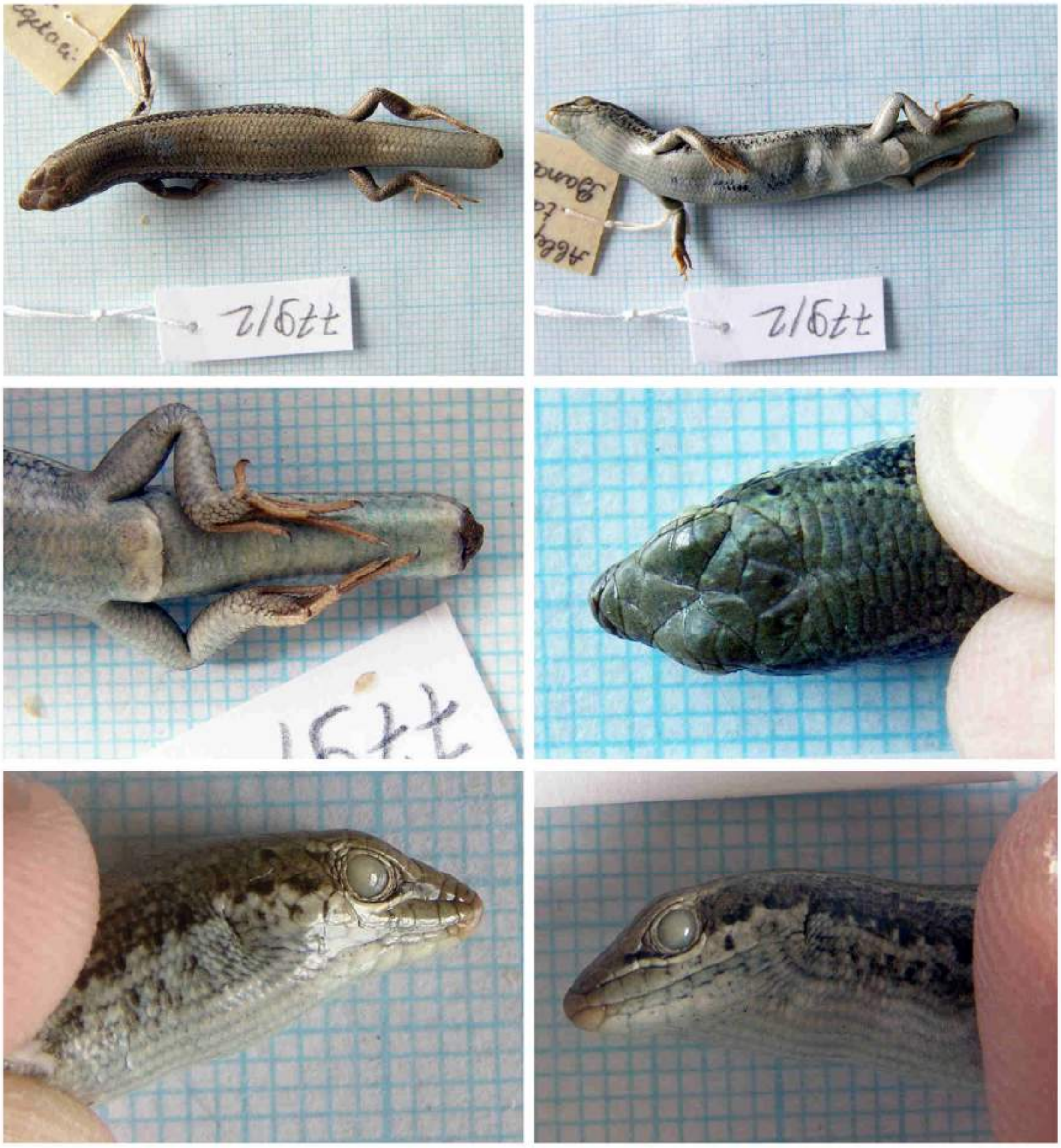

Supplement: Supplementary material 1 [file zookeys-843-129-s001.pdf]
